# Tumor-Induced PDPN^+^ Lymphatic-like Endothelial Cells Promote Clear-Cell Renal Cell Carcinoma Progression Through Reciprocal BMP10-CXCL13 Signaling

**DOI:** 10.3390/ijms27156994

**Published:** 2026-08-04

**Authors:** Tuong-Vi Nguyen, Hieu-Huy Nguyen-Tran, Thi-Ngoc Nguyen, Tien Hsu

**Affiliations:** 1 Department of Biomedical Sciences and Engineering, National Central University, 300 Zhongda Rd., Taoyuan City 320317, Taiwan; 108886602@cc.ncu.edu.tw; 2 Graduate Institute of Biomedical Sciences, China Medical University-Taiwan, 91 Hsueh-Shih Rd., Taichung 404333, Taiwan; nguyentranhieuhuy@mail.cmu.edu.tw (H.-H.N.-T.); ngocnguyen@mail.cmu.edu.tw (T.-N.N.)

**Keywords:** clear-cell renal cell carcinoma, lymphatic endothelial cell, VHL, CXCL13, BMP10

## Abstract

Here, we report the identification of a previously unrecognized population of tumor-induced podoplanin-positive (PDPN^+^) cells in clear-cell renal cell carcinoma (ccRCC) that exhibit features of under-differentiated lymphatic endothelial cells (LECs). These PDPN^+^ cells lack a complete repertoire of canonical LEC markers, including VE-cadherin, LYVE1, and VEGFR3, and fail to form functional lymphatic vessels, indicating a dysplastic phenotype. We termed these cells dysLECs and found that these cells are induced by BMP10 produced specifically by tumor cells. In turn, dysLECs secrete CXCL13, which promotes tumor cell proliferation and metastasis. Ligand-receptor analyses revealed a highly tumor-specific reciprocal signaling circuit: kidney tubule cells deficient in the von Hippel-Lindau (*VHL*) tumor suppressor gene uniquely express BMP10, a TGF-β family cytokine, whereas its receptor ALK1 is restricted to dysLECs; conversely, dysLECs produce CXCL13, while *VHL* mutant kidney tubule cells uniquely express its receptor, CXCR5. Pharmacological inhibition of ALK1 reduced CXCL13 production and suppressed the hyperplastic phenotype of *VHL* mutant tumor cells in vivo, whereas BMP10 neutralization inhibited tumor growth and metastasis in an orthotopic ccRCC xenograft model. Collectively, these findings identify a dysplastic population of PDPN^+^ lymphatic-like endothelial cells and define a tumor-specific BMP10-CXCL13 signaling axis that drives ccRCC progression, uncovering a previously unrecognized therapeutic vulnerability in this disease.

## 1. Introduction

Clear-cell renal cell carcinoma (ccRCC) is the most common subtype of renal cancer [1,2]. Genetically, ccRCC is closely linked to the inactivation of the von Hippel-Lindau (*VHL*) tumor suppressor gene, which is observed in up to 80% of sporadic cases [3,4]. Loss of *VHL* leads to constitutive activation of hypoxia-inducible factors (HIFs), resulting in abnormal transcriptional programs that promote angiogenesis, metabolic reprogramming, and immune evasion [5,6]. Although extensive research has clarified the downstream effects of *VHL* deficiency in established tumors, the molecular and cellular events that reshape the tumor-permissive microenvironment are still not completely elucidated. Increasing evidence has shown that components of the tumor microenvironment, including fibroblasts, immune cells, blood vessels, and the lymphatic system, can support or suppress tumor progression [7,8]. Although angiogenic and immune responses induced by ccRCC tumor cells have been extensively studied [5,9], the interactions between *VHL*-deficient epithelial cells and lymphatic endothelial cells (LECs) remain poorly understood.

The lymphatic system is vital for fluid homeostasis, immune surveillance, and metabolic regulation [10]. It helps maintain the balance of interstitial fluid, absorbs dietary lipids, and facilitates the movement of immune cells [11,12,13]. In the context of cancer, the tumor-associated lymphatic system is the route for metastatic spread but is also the site of tumor antigen presentation [14]. As such, therapeutics against the tumor-associated lymphatic system, although therapeutically accessible, have not been widely attempted because of the dual roles of the lymphatic system in cancer progression. Therefore, a unique prooncogenic pathway in the tumor-associated lymphatic system may provide a valuable therapeutic target.

We have developed a genetically engineered mouse model that selectively deletes [knockout (KO)] *Vhlh* (*VHL-homology* locus, the mouse *VHL* allele) in subpopulations of renal tubular epithelial cells (see Section 4). This model recapitulates the key features of early-stage ccRCC, such as epithelial piling-up, hyperplasia, cyst formation, hyper-angiogenesis, and inflammatory remodeling [6,15]. It is, therefore, a useful autochthonous model for premalignant ccRCC [16]. We observed significant morphological changes in the lymphatic vasculature during the analysis of the pathological features of this mouse model. This study uncovered a podoplanin^+^ (Pdpn^+^) cell population with under-differentiated lymphatic endothelial cell characteristics specific to the tumor tissue, and elucidated a previously unrecognized BMP10-CXCL13 reciprocal signaling axis that induces the formation of these cells and ultimately promotes the progression of ccRCC. Targeting this tumor-specific group of cells should avoid the potential undesirable side effects of targeting the functional lymphatic vessels.

## 2. Results

### 2.1. ccRCC-Associated Lymphatics Are Disorganized and Enriched with Pdpn^+^ Cells

**Figure 1:**
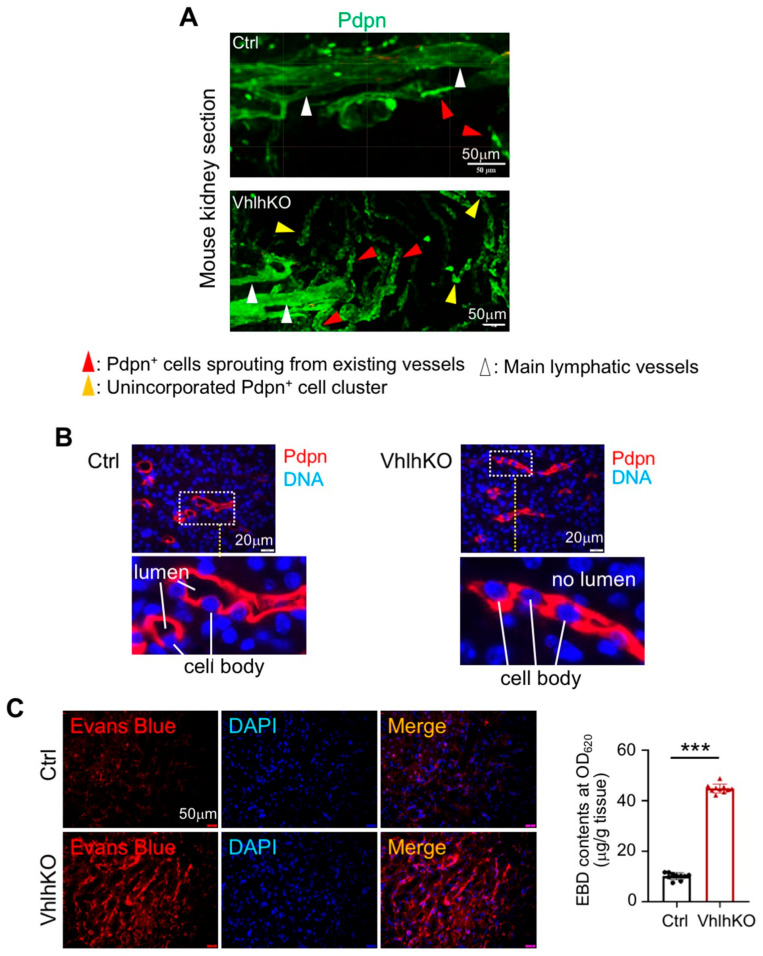
The lymphatic system associated with VhlhKO kidney tissue is disorganized. (**A**): Podoplanin (Pdpn) marker staining shows a disorganized lymphatic system in the VhlhKO mouse compared with Control (Ctrl). (**B**): Kidney sections from Ctrl and VhlhKO mice were stained for Pdpn and nucleus (DAPI). Pdpn^+^ cells in Ctrl form tubular structures with lumen, indicating functional vasculature, while Pdpn^+^ cells in VhlhKO form linear arrays of cells without lumen formation. (**C**): Kidneys of Ctrl and VhlhKO mice were injected with Evans Blue dye (EBD). The kidneys were excised 30 min later. The retained EBD (red) in Ctrl and VhlhKO kidneys was visualized by immunofluorescence (540 nm excitation). The total amount of EBD was extracted and measured by optical density (OD) at 620 nm (right panel). VhlhKO samples show a significant amount of retained EBD, indicating impaired lymphatic drainage capacity. Scale bars are 50 μm. Statistical analysis was performed by Student’s *t*-test. *n* = 10. ***, *p* < 0.001. Each data point represents one independent animal.

To evaluate the integrity of the lymphatic system in kidney tissues containing *VHL*-deficient tubule cells, we examined the expression of lymphatic cell markers Lyve1, Vegfr3/Flt4, and Pdpn [17], comparing the control (Ctrl; *Hoxb7-Cre-GFP*/+) and VhlhKO (*Hoxb7-Cre-GFP*/*+*; *Vhlh^loxP/loxP^*) kidneys. Interestingly, only with Pdpn did we observe that the lymphatic system in the VhlhKO mouse kidney was disorganized, with abnormal sprouting and clusters of unincorporated cells, in comparison with the Ctrl counterpart, which showed an organized array of lymphatic vessels (Figure 1A). Significantly, clusters of unincorporated Pdpn^+^ cells were observed that did not form tubule structures but formed linear arrays of cells without lumen, as compared with the normal Pdpn^+^ tubular structures with lumen found in Ctrl tissue (Figure 1B).

The disorganized lymphatic system and appearance of unincorporated Pdpn^+^ cell clusters imply impaired lymphatic functions. Indeed, the interstitial drainage capacity measured by the clearance of injected dye was significantly reduced in the VhlhKO kidney (Figure 1C).

### 2.2. Increased Numbers of PDPN^+^-Expressing Cells in VHL-Deficient Kidney Tissues

**Figure 2:**
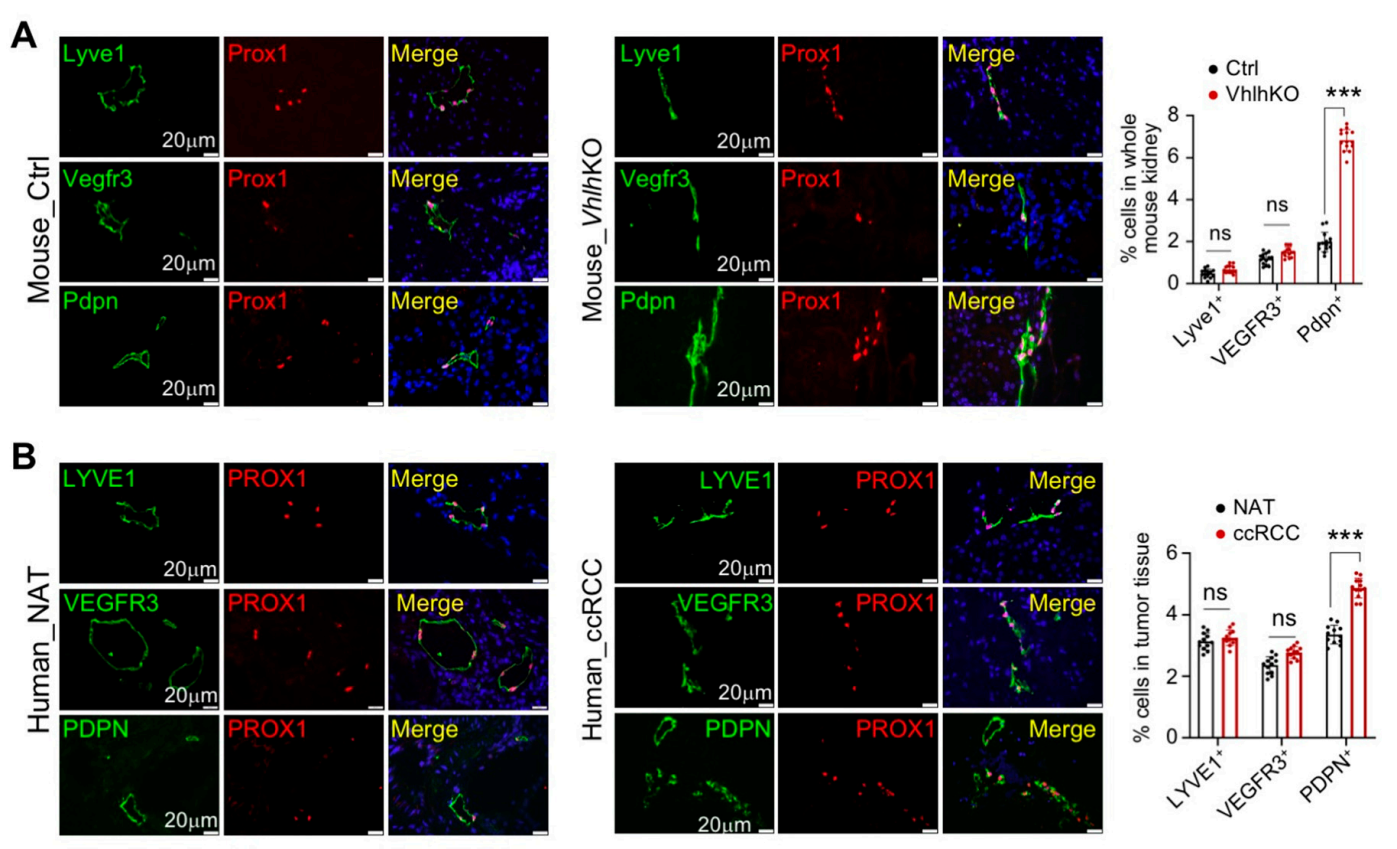
*VHL/Vhlh*-deficient kidneys display a specific increase in the number of PDPN^+^ cells. (**A**): Immunofluorescence staining of kidney sections showing expression of Pdpn, Lyve1, or Vegfr3 with Prox1 in Ctrl and VhlhKO mice (*n* = 14). There is a specific increase in the number of Pdpn^+^ cells, but not Lyve1^+^ or Vegfr3^+^ cells. Scale bars are 20 μm. ns, no significance; ***, *p* < 0.001. Each data point represents one independent animal. (**B**): Immunofluorescence staining of ccRCC tumor sections showing co-expression of PDPN, LYVE1, or VEGFR3 with PROX1 in normal adjacent tissue (NAT) and ccRCC tumor tissue (*n* = 12). There was a specific increase in the number of PDPN^+^ cells, but not LYVE1^+^ and VEGFR3^+^ cells, in ccRCC tissues compared with NAT. Scale bars, 20 μm. ns, no significance; ***, *p* < 0.001. Each data point represents one independent sample.

To further analyze the PDPN-specific phenotype of the lymphatic system in *VHL*-deficient tissues, we compared the expression of three commonly used lymphatic cell markers—LYVE1, VEGFR3, and PDPN—co-stained for the pan-lymphatic transcription factor PROX1 to verify the lymphatic vessel identity [18]. As shown in Figure 2, in both VhlhKO mouse kidney (Figure 2A) and ccRCC patient samples (Figure 2B), LYVE1^+^ and VEGFR3^+^ lymphatic cell numbers remained the same in *VHL*-deficient tissues and in their respective normal tissue counterparts [Ctrl in mouse and normal adjacent tissue (NAT) in human ccRCC], whereas PDPN^+^ cell numbers were significantly increased in *VHL*-deficient tissues compared with their normal counterparts. This indicates that LYVE1 and VEGFR3 are associated with functioning lymphatic vessels in both normal and *VHL*-deficient tissues, whereas abnormal PDPN^+^ cells are induced by *VHL*-deficient epithelial cells and may constitute the disorganized lymphatic vasculature.

Interestingly, the percentages of these Pdpn^+^ cells co-expressing other lymphatic endothelial cell markers, including VE-cadherin, Lyve1, and Vegfr3, were significantly reduced in VhlhKO kidney compared with Ctrl (Figure S2). Therefore, the majority of these tumor-associated PDPN^+^ cells are not fully differentiated or are dedifferentiated lymphatic endothelial cells. We designate these cells dysplastic lymphatic endothelial cells (dysLECs).

### 2.3. ccRCC-Associated PDPN^+^ Dysplastic Lymphatic Endothelial Cells (dysLECs) Overexpress CXCL13

**Figure 3:**
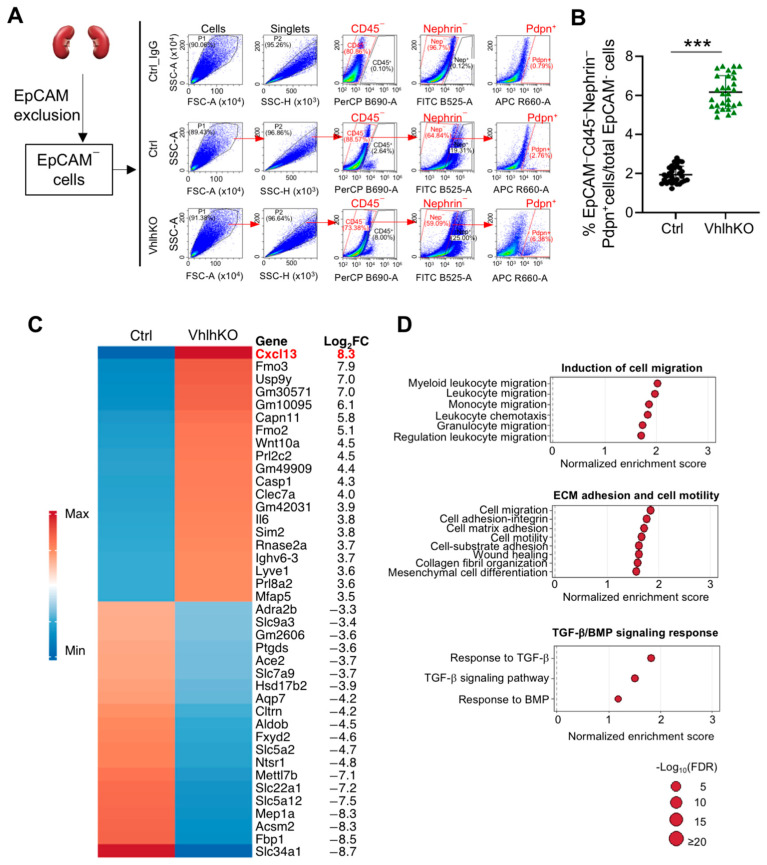
Pdpn^+^ dysplastic lymphatic endothelial cells (dysLECs) display gene expression patterns indicating increased motility, and Cxcl13 is selectively upregulated in *Vhlh*-deficient kidneys and human ccRCC. (**A**): Cell sorting strategy for EpCAM^−^Pdpn^+^Cd45^−^Nephrin^−^ cells from Ctrl or VhlhKO kidney. (**B**): The EpCAM^−^Cd45^−^Nephrin^−^Pdpn^+^ cells are much more numerous in VhlhKO compared with those in Ctrl. *n* = 30. ***, *p* < 0.001. Each data point represents one independent sample. (**C**): Heatmap of transcriptomes comparing EpCAM^−^Cd45^−^Nephrin^−^Pdpn^+^ cells from Ctrl and VhlhKO mouse kidneys (Supplementary Data S2), showing the top 20 most upregulated and downregulated genes. (**D**): Gene set enrichment analysis of biological processes identifies pathways related to induction of cell migration, ECM adhesion, cell motility, and TGF-β/BMP signaling response.

Next, we further characterized these Pdpn^+^ dysLECs by isolating them for molecular analysis. We first removed EpCAM^+^ epithelial cells from the whole kidney cell suspension [19]. EpCAM was selected for epithelial-cell enrichment because it is a well-established cell-surface epithelial marker that enables antibody-based isolation of viable cells and has been used extensively to identify or purify renal epithelial populations from dissociated kidney tissue. Then, to exclude certain myeloid cells and podocytes that are known to also express Pdpn [20,21], we isolated primary EpCAM^−^CD45^−^Nephrin^−^Pdpn^+^ cells from Ctrl and VhlhKO mouse kidneys (Figure 3A). There was a significant increase in the CD45^−^Nephrin^−^Pdpn^+^ population in the EpCAM^−^ population of kidney cells in VhlhKO compared with the Ctrl (Figure 3B), consistent with the tissue IHC results (Figure 2A).

Since some fibroblastic cells, including pericytes, can express Pdpn, we verified using qRT-PCR that the EpCAM^−^CD45^−^Nephrin^−^Pdpn^+^ cells express LEC markers such as *Pdpn*, *Lyve1*, and *Vegfr3 (Flt4)*, but not fibroblastic cell markers such as *αSMA*, *Col1a1*, and *Pdgfrb* (Figure S3; Supplementary Data S1). Note that the qRT-PCR assay quantified the amount of a specific RNA species per unit total RNA, not per cell. Therefore, the differential expression of *Pdpn* shown in qRT-PCR is not as pronounced as in tissue IHC, which quantified the number of cells expressing a specific gene product. Furthermore, we showed that in VhlhKO tissue, Pdgfrb^+^ cells can be closely associated with irregular Pdpn^+^ lymphatic vessels but are separate from these Pdpn^+^ cells (Figure S4).

We then performed bulk RNA sequencing (RNA-Seq) on these primary dysLECs to gain better insights into the functions of these cells. Differential gene expression analysis showed that EpCAM^−^CD45^−^Nephrin^−^Pdpn^+^ LECs from VhlhKO and Ctrl kidneys exhibited very different transcriptomes (Supplementary Data S2). The top 20 most overexpressed and most downregulated genes in VhlhKO are shown in a heatmap (Figure 3C). The most highly overexpressed gene encodes the chemokine *Cxcl13* (Figure 3C). Biological process analysis (Figure 3D) indicated that the Pdpn^+^ LECs in VhlhKO are enriched for genes involved in induction of cell migration, ECM adhesion, and cell motility, and interestingly, TGF-β/BMP signaling. This indicates that the Pdpn^+^ dysLECs exhibit mesenchymal phenotypes (increased motility and response to TGF-β/BMP signaling) and can induce cell motility of other cells (via chemokine Cxcl13).

**Figure 4:**
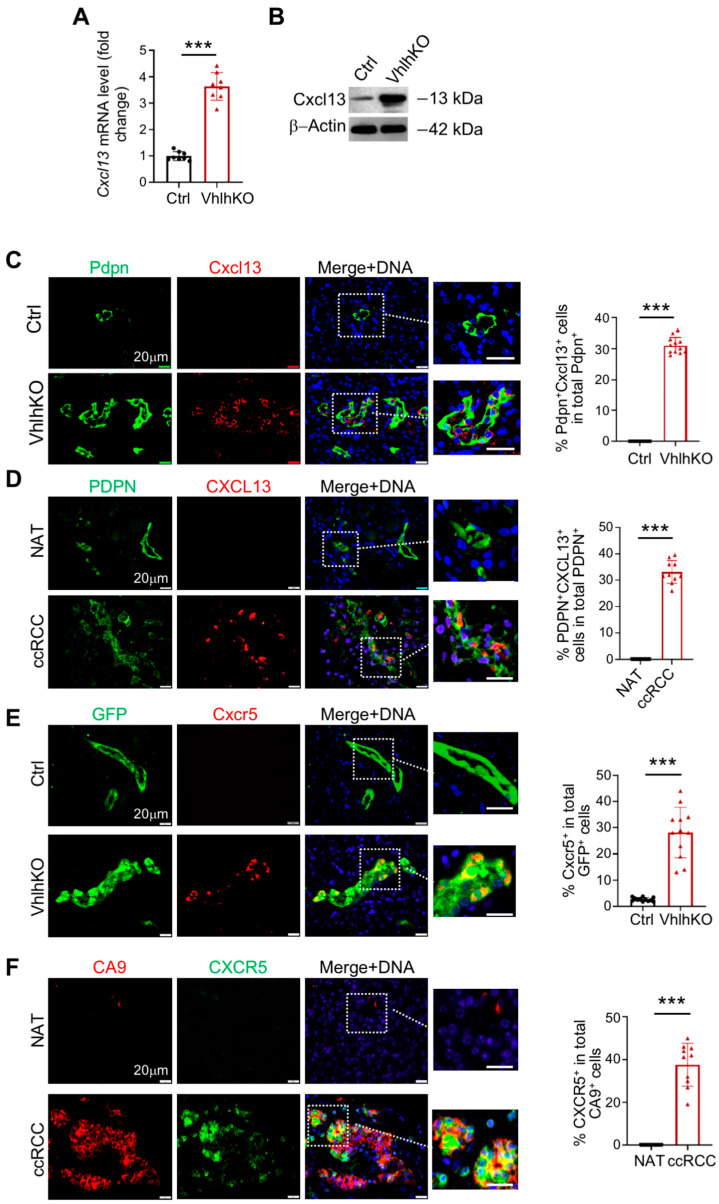
Ligand-receptor pair CXCL13-CXCR5 is specifically expressed in PDPN^+^ dysLECs and *Vhlh/VHL*-deficient epithelial/tumor cells, respectively. (**A**): qRT-PCR validation of *Cxcl13* overexpression in VhlhKO EpCAM^−^Cd45^−^Nephrin^−^Pdpn^+^ dysLECs. ***, *p* < 0.001. (**B**): Protein blot validation of Cxcl13 overexpression in VhlhKO EpCAM^−^Cd45^−^Nephrin^−^Pdpn^+^ dysLECs. (**C**): Kidney sections from Ctrl (*Hoxb7-Cre-GFP*/+) and VhlhKO (*Hoxb7-Cre-GFP*/+*; Vhlh^loxP^*^/*loxP*^) mice were stained for Pdpn and Cxcl13. There is no expression of Cxcl13 in Pdpn^+^ cells in the Ctrl, while Pdpn^+^Cxcl13^+^ cells were significantly increased in VhlhKO. Scale bars are 20 μm. Quantification of the percentage of Pdpn^+^Cxcl13^+^ cells was performed using the CellProfiler software v4.2.8 (used on 5 May 2026). *n* = 12. ***, *p* < 0.001. Each data point represents one independent animal. (**D**): Kidney sections from NAT and ccRCC tissues were stained for PDPN and CXCL13. There is no expression of CXCL13 in PDPN^+^ cells in NAT; whereas, PDPN^+^CXCL13^+^ cells were significantly increased in ccRCC tissue. Scale bars are 20 μm. Quantification of the percentage of Pdpn^+^Cxcl13^+^ cells was performed using the CellProfiler software. *n* = 10. ***, *p* < 0.001. Each data point represents one independent sample. (**E**): Kidney sections from Ctrl and VhlhKO mice were stained for GFP (a marker for *Vhlh* KO cells and their Ctrl counterparts) and Cxcr5. There is very little Cxcr5 in Ctrl GFP^+^ cells, while GFP^+^Cxcr5^+^ cells are significantly increased in VhlhKO. Scale bars are 20 μm. Quantification of the percentage of Cxcr5^+^ in total GFP^+^ cells was performed by the CellProfiler software. *n* = 12. ***, *p* < 0.001. Each data point represents one independent animal. (**F**): Kidney sections from NAT and ccRCC tissues were stained for CA9 (a ccRCC tumor cell marker) and CXCR5. There were very few CA9^+^ cells in NAT and no CXCR5 expression, whereas CA9^+^ cells were prevalent in ccRCC tissue and CA9^+^CXCR5^+^ cells were significantly increased. Scale bars are 20 μm. Quantification of the percentage of CXCR5^+^ in total CA9^+^ cells was performed using the CellProfiler software. *n* = 10. ***, *p* < 0.001. Each data point represents one independent sample.

The expression of the *Cxcl13* gene was confirmed in the primary EpCAM^−^Cd45^−^Nephrin^−^Pdpn^+^ cells (Figure 4A,B). In vivo, Cxcl13 protein was overexpressed in VhlhKO-associated Pdpn^+^ cells in tissue sections, whereas no Cxcl13 expression was detected in Ctrl tissue (Figure 4C). This is also validated in human ccRCC samples, as CXCL13 was co-expressed with PDPN^+^ cells in ccRCC tissue, but was not expressed at all in NAT (Figure 4D).

Furthermore, the only known CXCL13 receptor CXCR5 [22] is almost exclusively expressed in *Vhlh/VHL*-deficient epithelial cells in VhlhKO mice (Figure 4E) and tumor cells in ccRCC tissue (Figure 4F), suggesting a highly cell type-specific oncogenic pathway upregulated in dysLECs is endothelial cell-specific. We, therefore, investigated the BMP10 signaling further.

### 2.4. PDPN^+^ Dysplastic Lymphatic Endothelial Cells (dysLECs) Exhibit Increased BMP10 Signaling That Promotes the Prooncogenic Activity

**Figure 5:**
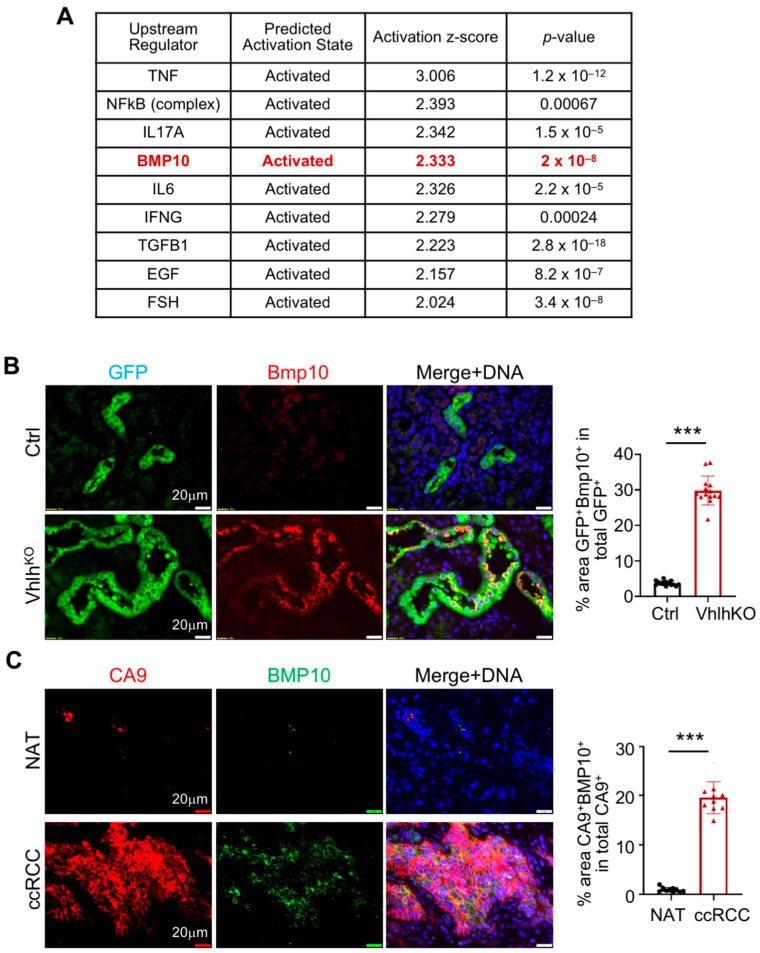
BMP10 signaling mediates activation of lymphatic endothelial cells (LECs) by *Vhlh/VHL*-deficient cells in mouse and human cells. (**A**): Upstream regulator analysis of RNA-Seq-identified differentially expressed genes in Pdpn^+^ dysLECs from VhlhKO vs. Ctrl. BMP10 signaling is notable for its specific role in endothelial development during embryogenesis. (**B**): Kidney sections from Ctrl (*Hoxb7-Cre-GFP*/+) and VhlhKO (*Hoxb7-Cre-GFP*/+*; Vhlh^loxP^*^/*loxP*^) mice stained for GFP and BMP10. There is very little BMP10 expression in the Ctrl compared with the significant expression of BMP10 specifically in *Vhlh*-deficient cells (GFP^+^). Quantification of the percentage area of Bmp10^+^ was performed using the CellProfiler software. *n* = 14. ***, *p* < 0.001. Each data point represents one independent sample. (**C**): Sections from NAT and ccRCC tissue stained for CA9 (a ccRCC tumor cell marker) and BMP10. There is little CA9 and BMP10 expression in NAT compared with the significant expression of BMP10 specifically in ccRCC tumor cells (CA9^+^). Quantification of the percentage area of BMP10^+^ was performed by the CellProfiler software. *n* = 10. ***, *p* < 0.001. Each data point represents one independent sample.

To identify the signaling pathway that activates the *VHL*-deficient cell-associated Pdpn^+^ dysLEC cell fate, an upstream regulator analysis was conducted using the Ingenuity Pathway Analysis (IPA) algorithm of the transcriptome of VhlhKO-associated EpCAM^−^Cd45^−^Nephrin^−^Pdpn^+^ dysLECs. Several signaling pathways of growth factors and cytokines were identified (Figure 5A), among which BMP10 is notable for its involvement in endothelial cell fate determination during embryogenesis and in embryonic and prenatal vascular and lymphatic remodeling [23,24]. It is normally not present in most adult tissues except in the heart [25]. None of the other signaling pathways were upregulated, consistent with the potential involvement in tumor-associated dysLEC activation. Bmp10 was extensively expressed in VhlhKO cells (GFP^+^) but not in their Ctrl counterparts (Figure 5B). Similarly, BMP10 was extensively expressed in *VHL*-deficient ccRCC cells (CA9^+^) but not in NAT (Figure 5C). This implies that BMP10 signaling in dysLECs is induced by BMP10 emanated from *VHL*-deficient tumor cells. We then tested whether BMP10 expression is directly linked to *VHL* inactivation in kidney tubule cells. Immortalized non-cancerous human kidney tubule cells were used with (HK-2^VHLKO^) or without (HK-2^WT^) *VHL* KO as an early-stage ccRCC tumor cell model. We verified that in the *VHL*-KO human kidney tubule epithelial cell line HK-2 (HK-2^VHLKO^), BMP10 was significantly overexpressed compared with the *VHL*-WT control (HK-2^WT^; Figure S5). In addition, the BMP10 overexpression in HK-2^VHLKO^ was partly HIF-dependent, as treatment with an HIF inhibitor Acriflavin (Acri) could reduce the extent of BMP10 overexpression (Figure S5).

BMP10 belongs to the TGF-β family of growth factors. Its receptor complex consists of the type 1 receptor activin receptor-like kinase 1 (Alk1; or Acvrl1), which is shared with the other endothelial cell-specific BMP, BMP9 [26], as well as the type 2 receptor BMPR2 and coreceptor Endoglin. BMP9 and BMP10 share a high degree of structural similarity; however, BMP9 has been shown to inhibit lymphatic development [27]. Therefore, it is significant that the upstream regulator analysis identified BMP10 signaling, but not BMP9 signaling (Figure 5A).

**Figure 6:**
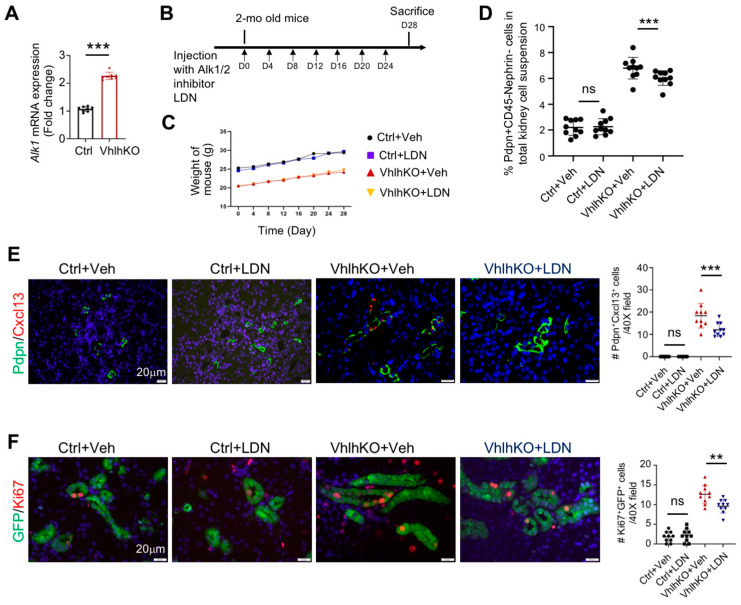
Alk1/2 inhibition reduces Cxcl13 expression and proliferation of *Vhlh*-deficient cells in vivo. (**A**): Isolated EpCAM^−^Cd45^−^Nephrin^−^Pdpn^+^ cells were processed for qRT-PCR analysis. There is a significant increase in the BMP10 type 1 receptor *Alk1* mRNA level in EpCAM^−^Cd45^−^Nephrin^−^Pdpn^+^ cells from VhlhKO kidney compared with the Ctrl. *n* = 8. ***, *p* < 0.001. Each data point represents one independent sample. (**B**): 2-month-old Ctrl or VhlhKO mice were injected with Alk1/2 inhibitor LDN-212854 (LDN) intraperitoneally at a dosage of 9 mg/kg or DMSO (Vehicle; Veh) every four days for a duration of 24 days. The mice were sacrificed and the kidneys excised on Day 28. (**C**): Mice were monitored for weight gain or loss as an indicator of general health. All experimental groups grew steadily at the same rate through the treatment regimen, although VhlhKO mice had a congenitally lower body weight. (**D**): Kidney sections from the 4 treatment groups were resected, and the cell suspension was subjected to cell sorting analysis for the percentage of EpCAM^−^Cd45^−^Nephrin^−^Pdpn^+^ cells in total EpCAM^−^ cells. *n* = 10. ns, no significance; ***, *p* < 0.001. Each data point represents one independent sample. (**E**): Kidney sections from the 4 treatment groups were stained for Pdpn and Cxcl13. There is no Cxcl13 expression in Ctrl treated or untreated with LDN. Cxcl13 is significantly overexpressed in VhlhKO tissue, and the expression is reduced by LDN treatment. Quantification of the number of Pdpn^+^Cxcl13^+^cells was performed by CellProfiler software. *n* = 10. ns, no significance; ***, *p* < 0.001. Each data point represents the average of 5 random 40× fields of view of one independent sample. (**F**): Kidney sections from the 4 treatment groups were stained for GFP (for *Vhlh*-deficient cells and the Ctrl counterparts) and Ki67. Proliferation (Ki67 expression) is low in Ctrl and is not altered by LDN treatment, while there is a significant increase in proliferation in GFP^+^ cells (*Vhlh*-deficient) in VhlhKO, and the level was significantly reduced by LDN treatment. *n* = 10. ns, no significance; **, *p* < 0.01. Each data point represents the average of 5 random 40× fields of view of one independent sample.

We next examined whether BMP10 could induce dysLEC formation and tumor progression. Since there are currently no commercially available antibodies against mouse Alk1 suitable for immunohistochemistry, we verified that *Alk1* mRNA was enriched in isolated primary EpCAM^−^Cd45^−^Nephrin^−^Pdpn^+^ cells (Figure 6A). In the following in vivo experiment, we chose to use the specific Alk1/2 inhibitor LDN-212854 (LDN) (Figure 6B) instead of *Alk1* KO in the VhlhKO background because such double conditional KO necessitates the use of two tissue-specific *Cre* transgenes (for kidney tubule and LECs), which, in our experience, will result in unpredictable health problems. Constitutive KO is also not applicable because *Alk1* function is essential for embryonic vascular development and *Alk1* KO is embryonic lethal [28]. We showed that LDN treatment did not affect the general health of the treated Ctrl or VhlhKO mice, as indicated by the same steady rates of weight gain in both genotypes (Figure 6C), although VhlhKO mice had a congenitally lower body weight. Previously, we showed that these animals exhibited marked renal fibrosis and impaired kidney function, characterized by proteinuria [6]. Mitigating the renal inflammatory phenotypes can enhance both the general health and longevity of these mice. Therefore, while the precise systemic etiology of their compromised state of health remains under investigation, it is likely that their underlying renal pathology is a major contributing factor.

More importantly, LDN treatment could reduce the dysLEC population as measured by cell sorting analysis for EpCAM^−^Cd45^−^Nephrin^−^Pdpn^+^ cells (Figure 6D). This is consistent with the above notion that BMP10 signaling can induce the dysLEC cell fate. We verified that recombinant mouse BMP10 could induce the canonical Smad1/5/8 signaling in EpCAM^−^Cd45^−^Nephrin^−^Pdpn^+^ cells, and the induction was inhibited by anti-BMP10 neutralizing antibody and LDN (Figure S6).

Importantly, Cxcl13 expression level in Pdpn^+^ cells in VhlhKO kidney is reduced by LDN treatment (Figure 6E). Furthermore, inhibition of Alk1 can greatly reduce the proliferative index (Ki67 expression) of VhlhKO kidney tubule cells (Figure 6F).

### 2.5. CXCL13 Enhances Proliferative and Migratory Capacities of VHL-Deficient Renal Tubule Cells

Since CXCL13 belongs to the chemokine family of proteins, we next examined whether CXCL13 possessed inductive activity on *VHL*-deficient tumor cells. As shown in Figure S7A, treatment with recombinant human CXCL13 did not affect the proliferation index (Ki67 expression) and the growth rate of HK-2^WT^. In contrast, CXCL13 treatment significantly increased proliferation (Ki67 expression) and growth rate of HK-2^VHLKO^ cells. In addition, CXCL13 had no effect on the migration and invasion of HK-2^WT^ cells but could significantly induce these two indicators of cell motility in HK-2^VHLKO^ cells (Figure S7B,C). It should be noted that *VHL* inactivation alone can promote cell motility, and exogenous CXCL13 could further increase such motility in *VHL*-deficient cells.

### 2.6. BMP10 Activity Is Required for Malignant Progression of ccRCC

We next tested the therapeutic potential of BMP10 inhibition using a neutralizing anti-BMP10 antibody against xenografted ccRCC cells. This strategy takes advantage of the fact that BMP10 function is not required in adults [23,26,29,30]. Indeed, we have shown above that there is very low, if any, expression of BMP10 in normal kidney tissues in mice and humans (Figure 5B,C). The neutralizing antibody dosage followed the established protocol [31,32].

We first verified that BMP10 is not an autocrine growth factor. We observed that BMP10 and ALK1/2 show minimal to no expression in normal adjacent tissue. In ccRCC tissue, while the BMP10 receptor ALK1 was mostly absent from the tumor cells themselves (CA9^+^ cells), BMP10 is robustly expressed within the tumor cells (Figure S8). Therefore, it is unlikely that BMP10 can function as an autocrine growth factor.

We further verified that BMP10 was important for inducing and/or maintaining dysLECs. Ctrl and VhlhKO mice were treated with anti-BMP10 neutralizing antibody. We observed that anti-BMP10 treatment did not alter the number of Lyve1^+^ and Vegfr3^+^ cells in either the Ctrl or VhlhKO kidneys. Notably, however, the treatment significantly reduced the number of Pdpn^+^ cells specifically in VhlhKO, with no effect in the Ctrl. These results support the hypothesis that BMP10 specifically induces the Pdpn-expressing dysLECs (Figure S9).

**Figure 7:**
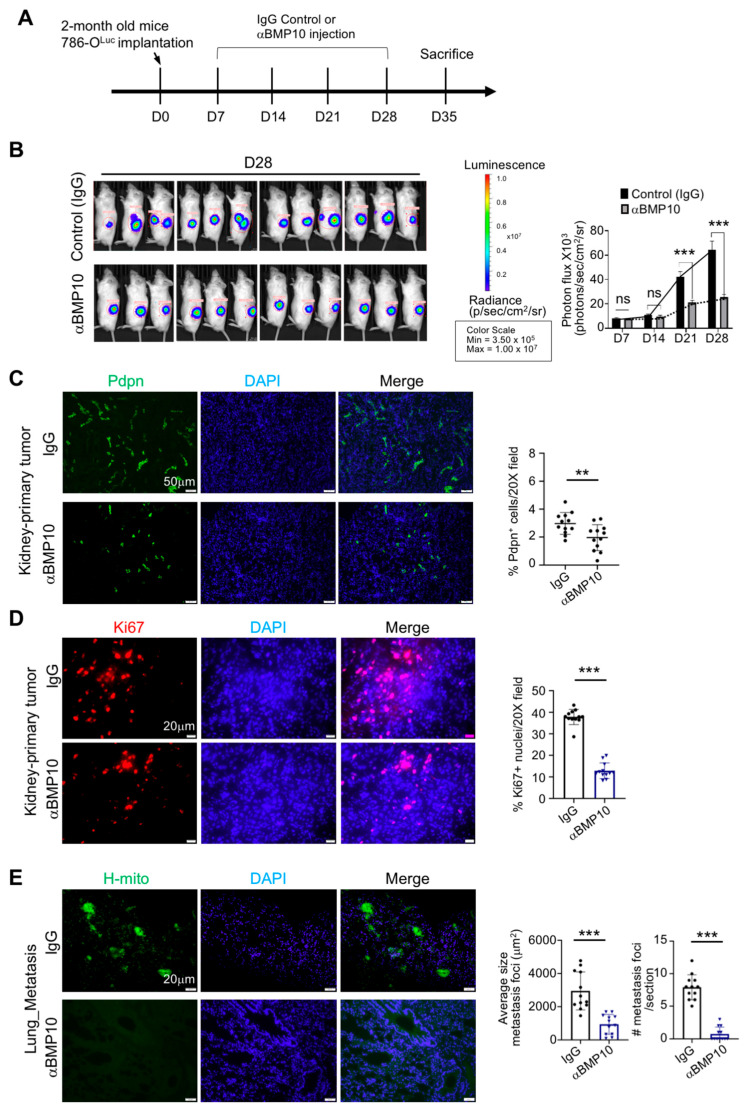
BMP10 inhibition suppresses tumor progression and metastasis in vivo. (**A**): Schematics of ccRCC cell implantation to the left kidney and IV injection of control IgG or anti-BMP10 neutralizing antibody (αBMP10). (**B**): IVIS examination of xenografted mice at designated times after cancer cell implantation. The growth inhibitory effect of αBMP10 becomes obvious on D21. *n* = 12. ns, no significance; ***, *p* < 0.001. Each data point represents one independent sample. (**C**): Kidney sections from sacrificed xenografted mice on D35 were stained for Pdpn. αBMP10 treatment significantly reduced the number of Pdpn^+^ cells of the primary tumor. Bars are 50 μm. *n* = 12. **, *p* < 0.01. Each data point represents the average of 5 random 20× fields of view of one independent sample. (**D**): Kidney sections from sacrificed xenografted mice on D35 were stained for Ki67. αBMP10 treatment significantly reduced proliferation of the primary tumor. Bars are 20 μm. *n* = 12. ***, *p* < 0.001. Each data point represents the average of 5 random 20× fields of view of one independent sample. (**E**): Lung sections from sacrificed xenografted mice on D35 were stained for human mitochondria (H-mito) to visualize metastasized ccRCC cells. αBMP10 treatment significantly reduced the size and number of metastatic foci. Bars are 20 μm. *n* = 12. ***, *p* < 0.001. Each data point represents the average of 5 random 20× fields of view of one independent sample.

To test the prooncogenic activity of BMP10, 2-month-old immune-deficient mice were implanted with 5 × 10^5^ ccRCC cells 786-O expressing luciferase (786-O^Luc^) into the subrenal capsular space. IgG (control) or anti-BMP10 antibody (αBMP10) was then injected via the tail vein from Day 7 after implantation every week for 3 weeks (a total of 4 injections) (Figure 7A). The mice were assessed for tumor growth weekly after every antibody injection using the in vivo imaging system (IVIS), and sacrificed on Day 35. As shown in Figure 7B, the primary xenografted tumors grew rapidly in IgG-treated mice after Day 14, and the growth was significantly reduced by anti-BMP10 antibody treatment on Day 21 and Day 28. The effect of anti-BMP10 therapy was verified by histological and pathological examination of the tumor tissue. Anti-BMP10 antibody treatment reduced the number of Pdpn^+^ cells (Figure 7C), consistent with the above observation that BMP10 signaling is required for Pdpn^+^ dysLEC formation. Importantly, the IgG-treated primary tumor showed strong Ki67 expression, which was suppressed by anti-BMP10 treatment (Figure 7D). Accordingly, both the number and the size of metastatic foci in the lung were reduced upon anti-BMP10 treatment (Figure 7E).

We have performed the anti-BMP10 therapeutic test using the xenograft model of a different ccRCC cell line A498. Anti-BMP10 treatment also inhibited Pdpn^+^ induction, tumor growth, and lung metastasis (Figure S10).

## 3. Discussion

**Figure 8:**
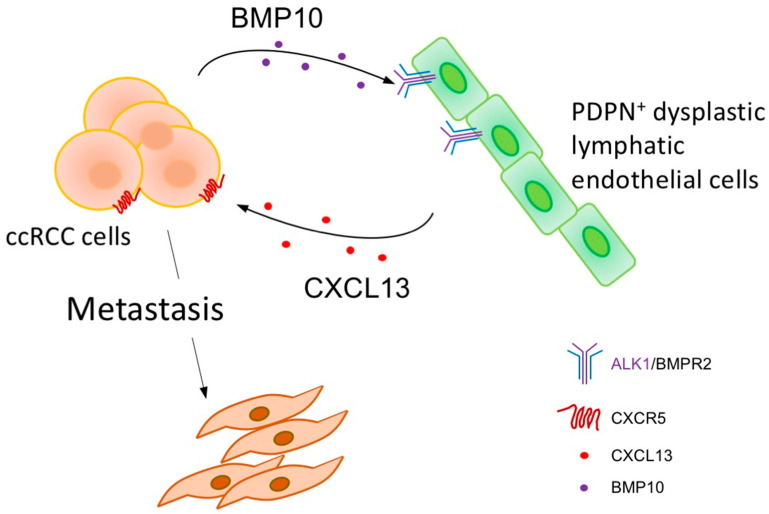
Reciprocal signaling of BMP10 and CXCL13 pathways in activation of ccRCC-associated lymphatic endothelial cells and tumor progression.

In this study, we identified a reciprocal signaling loop involving the BMP10 and CXCL13 pathways (summarized in Figure 8). We uncovered a previously uncharacterized group of PDPN^+^ cells, the majority of which co-expressed only some of the other canonical lymphatic cell markers, such as VE-cadherin, Lyve1, or Vegfr3 (Figure S2). They do not form functional lymphatic vessels but form linear arrays of cell clusters without lumen (Figure 1B). Therefore, they are likely either undifferentiated LECs or dedifferentiated LECs. Indeed, PDPN^+^ lymphatic endothelial progenitor cells have been identified previously and can differentiate into LYVE1-expressing mature LECs [33,34]. Future studies should verify the origin of the dysLECs identified in this study and determine whether they are abnormal lymphatic progenitor cells.

Regardless of their origin, we discovered that these dysLECs exhibit specific and potent prooncogenic functions that are pharmacologically targetable.

We chose to focus on Cxcl13 because it is the highest-expressing gene in the dysLEC, and we show that the CXCL13-CXCR5 signaling axis is specifically expressed by dysLEC and *VHL*-deficient epithelial cells, respectively (Figure 4). However, we should note that other genes overexpressed in dysLECs, such as Wnt10a and IL6, can also contribute to tumor cell migration (Figure 3C).

As well, we chose to focus on BMP10 signaling because it is an endothelial cell-specific signaling system, thus presenting the best option as a therapeutic target. Other signaling pathways identified in Figure 5A, such as TNF, IL17A, IL6, and IFNG, may regulate the immune-modulating activity known to be present in the lymphatic endothelial cells, and NFκB and TGF-β may contribute to the endothelial-to-mesenchymal transition phenotype of these dysLECs.

Significantly, we showed that CXCL13 is specifically expressed by the BMP10-stimulated PDPN^+^ dysLECs and its sole receptor CXCR5 is specifically expressed by tumor cells (Figure 4). Reciprocally, BMP10 is specifically produced by tumor cells (Figure 5), and the receptor Alk1 is overexpressed in a *VHL*-deficient tissue-associated subpopulation of PDPN^+^ cells (Figure 6A). Therefore, this intercellular signaling system is highly amenable to therapeutic intervention (summarized in Visual Overview).

We demonstrated that HIF-1/2 inhibition reduced the overexpression of BMP10 in *VHL*-deficient HK-2 cells. This is consistent with the idea that BMP10 expression is induced in the pseudohypoxic *VHL*-deficient cells, albeit that the inhibition is only partially effective (Figure S5). Interestingly, our analysis of the ENCODE and Ensembl databases did not identify canonical hypoxia-response elements (HREs) within the enhancer or the first intron of either the human or mouse BMP10 genes. However, it is well-established that HIF-α can activate transcription independent of direct HRE binding by interacting with other transcription factors, such as STAT3, and recruiting the p300/CBP co-activator complex [35]. Supporting this potential mechanism, our Ensembl analysis identified a STAT3 binding site within the BMP10 enhancer. Furthermore, we have previously demonstrated that inhibiting JAK1/2 activity—thereby suppressing STAT1/3 activation—ameliorates the pathological phenotypes in VhlhKO mice [15]. It is, therefore, possible that HIF and Stat1/3 cooperate in BMP10 gene activation. Future studies should address this possibility.

We show that CXCL13 is expressed in the Pdpn^+^ LECs associated with *VHL*-deficient tissues in vivo and in EpCAM^−^CD45^−^Nephrin^−^Pdpn^+^ dysLECs (Figure 4A–C), and the expression is dependent on Alk1 signaling (Figure 6E,F).

Notably, BMP10 is mainly involved in embryonic vascular remodeling and lacks the normal essential adult function [23,26,29,30]. Its role in other physiological and pathological conditions has not been extensively studied. Some studies have suggested a growth inhibitory role of BMP10 in some cancers, although most of these presumed tumor suppressor activities are performed in vitro with a monoculture of established cancer cell lines [36,37].

The PDPN^+^ dysLECs produce CXCL13 that can induce cell motility (Figure S7). CXCL13 is a chemokine that attracts B lymphocytes to secondary lymphoid organs [38,39], and is involved in organizing the inflammatory responses in human diseases [39]. CXCR5 is the only known receptor for CXCL13, first identified in Burkitt’s lymphoma cell lines and lymphatic tissue [22]. This function may not be operable in ccRCC since we did not detect CXCR5 expression in the immune cell population in *VHL*-deficient tissues (Figure 4). It is, therefore, interesting that this highly specific ligand-receptor pair is hijacked by ccRCC for the development of malignancy.

It has been demonstrated that increased HIF activity in *VHL*-deficient tumor cells can induce the tumor cell-intrinsic epithelial-to-mesenchymal transition program, including increased migratory capacity [40]. This has been recapitulated in our data (Figure S7). It has also been recognized that the direct HIF-regulated pathways are necessary but insufficient for malignant tumor progression [3]. Here we show that CXCL13 signaling is another critical factor. As such, the reciprocal signaling mechanism can be leveraged for ccRCC therapy. Indeed, we have demonstrated in this study that an Alk1/2 inhibitor can reduce early hyperplasia in a premalignant autochthonous ccRCC model (Figure 6), and a neutralizing BMP10 antibody can ameliorate primary tumor growth and metastasis in an orthotopic ccRCC xenograft model (Figure 7). Thus, BMP10 as a tumor-specific secreted factor represents a desirable therapeutic target.

We should note that there is no direct demonstration of the presence of chemokines (BMP10 and Cxcl13) and chemokine receptors (Alkt1 and CXCR5) in the xenograft system. However, we show the following: (1) BMP10 expression is significantly upregulated by HIF in the kidney tubule cells (Figure S5), implicating that 786-O and A498, both highly expressing HIF-2α, can express BMP10. (2) In the xenograft analysis, mouse Pdpn-expressing, abnormal lymphatic cells (dysLECs) do appear within the primary tumors (Figure 7 and Figure S10), indirectly supporting the notion that the ccRCC cells can reconstitute the tumor microenvironment. (3) In clinical ccRCC tissues, the tumor cells express CXCR5 and BMP10 receptors (Figure 4F and Figure 5C), while in mice, Pdpn^+^ lymphatic cells express Cxcl13 and Alk1 (Figure 4C and Figure 6A).

We also note that Alk1/2 inhibition only caused partial reduction of the EpCAM^−^CD45^−^Nephrin^−^Pdpn^+^ cells (Figure 6D). The mild reduction of Pdpn^+^ cells in the CD45^−^Nephrin^−^Pdpn^+^ population may be because the BMP10-Alk1 signaling can alter the activity of Pdpn^+^ dysLECs, but not the Pdpn expression itself. Alternatively, the modest reduction of Pdpn-expressing cells observed in the total EpCAM^−^CD45^−^Nephrin^−^Pdpn^+^ population indicates that Alk1/2 inhibitor treatment selectively targets a specific subset of these cells.

These findings also suggest that while dysLECs represent a minority fraction within the broader renal lymphatic environment, they likely exert a disproportionately significant influence on ccRCC development and pathological lymphangiogenesis. This is a critical observation regarding the functional heterogeneity of the tumor microenvironment.

The lymphatic system is responsible for transporting antigen-presenting cells—macrophages and dendritic cells—from tissue parenchyma to lymph nodes to mount immune responses involving regulatory and effector cells [41,42]. This system also serves as a route for immune suppressor cells required for cancer cell-mediated immune evasion. In addition, lymphatic vessels are responsible for absorbing excessive protein and fluid from the interstitium and returning them to the blood circulation [11]. Since the lymphatic system is a part of the circulation conduit, it is also an accessible route for cancer cell metastasis. Therefore, because of the dichotomous functions of the lymphatic system in cancer progression [43], simple inhibition of lymphangiogenesis and lymphatic vascularization is unlikely to be a suitable therapeutic approach. Our discovery that anti-BMP10 signaling can specifically inhibit the action of PDPN^+^ dysLECs on tumor growth and metastasis offers a potentially important therapeutic strategy.

## 4. Materials and Methods

### 4.1. Reagents

Details of the reagents, media, and supplements are shown in Table S1, and all the primary and secondary antibodies used are shown in Table S2.

### 4.2. Experimental Animals

The original *Vhlh^loxP^*^/*loxP*^ strain (*Vhlh* is the mouse *VHL* genomic locus) was described previously [44] and obtained from the Jackson Laboratory. The *Hoxb7-Cre-GFP*; *Vhlh^loxP^*^/*loxP*^ strain with exon 1 deletion in the C57BL/6 background was generated by intercrossing *Hoxb7-Cre-GFP*/+; *Vhlh^loxP^*^/+^ mice. Littermates carrying the *Hoxb7-Cre-GFP*/+ genotype were used as controls. The *Hoxb7-Cre-GFP* driver line expresses Cre recombinase under the control of the *Hoxb7* promoter and includes a *GFP* reporter for *Cre* activity [15]. While *Hoxb7* is primarily recognized for its expression in the collecting duct during embryonic development [45], we have demonstrated *Hoxb7* promoter-driven reporter expression in other segments of the neonatal and adult renal tubule system. They include Lotus tetragonolobus lectin (LTL)-stained proximal tubules and Tamm-Horsfall protein (THP)-expressing thick ascending limbs [15]. This KO strain exhibits premalignant ccRCC phenotypes predominantly within THP-expressing cells [15], aligning with the seminal clinical findings that *VHL*-deficient proximal tubule cells exhibit little proliferative potential while *VHL*-deficient THP-positive tubule cells readily expand [46]. Phenotypic analyses of *Hoxb7-Cre-GFP*; *Vhlh^loxP^*^/*loxP*^ mice at 3 months of age exhibited 100% penetrance of cystic, hyperplastic, inflammatory, and angiogenic phenotypes [15]. For phenotypic analysis, male and female animals were used in a 2:1 ratio. The immune-deficient NOD/SCID mouse strain (*NOD.CB17-Prkdc^scid^*/*NcrCrl*; RRID:IMSR_CRL:394) was obtained from BioLASCO, Taiwan. Two-month-old male animals were used for xenograft experiments to minimize gender-specific influences since ccRCC exhibits a male-predominant bias [47]. The experiments ended at set time points and no human endpoints.

No confounders such as cage location or sex-independent weight differentials were noted during the experiments; therefore, no adjustments or exclusions were made. Group allocation was performed using random.org software 2026 Only the experimenter was aware of group allocation. Outcome assessment and data analysis were performed with persons unaware of the group allocation. Using the samplesz.xls spreadsheet, we calculated that for a given genotype, e.g., vascular abnormality that is 100% penetrant with standard deviation ~5–10% and estimated error ~10% based on previous studies [31,32], to have this be different from another genotype with a power of 95%, ~10 mice in each group are needed to have a 95% power of detecting this difference (*p*-value of 0.05).

The mice were housed in pathogen-free facilities with climate and light-cycle control. The cages were provided with nest-building materials and igloos for nesting. Adverse events were reported immediately. Anesthesia was used for all surgical procedures, and analgesia was administered post-operation. All experiments were performed in accordance with a protocol prepared before the study and were approved by the Institutional Animal Care and Use Committee of China Medical University (CMUIACUC-2024-034).

### 4.3. Tissue Dissociation and Cell Suspension

Ten-to-twelve-week-old male mice (in C57BL/6J background) were used, including *Hoxb7-Cre-GFP*/+ [Control (Ctrl)] and *Hoxb7-Cre-GFP*/+; *Vhlh^loxP^*^/*loxP*^ [*Vhlh* conditional KO (VhlhKO)], with 6 animals per group. The use of male mice was because ccRCC is a male-predominant disease [47]. The mice were anesthetized and perfused transcardially with 30 mL of Hank’s Balanced Salt Solution (HBSS; Gibco, Waltham, MA, USA; Cat #14025092) to remove circulating blood. Kidneys were excised and enzymatically dissociated in 10 mL of HBSS supplemented with 0.2% collagenase II (Sigma-Aldrich, Burlington, MA, USA; Cat. #6885), 0.04% Dispase (Sigma-Aldrich, Burlington, MA, USA; Cat. #D4693), 1 mg/mL DNase I (Merck, Darmstadt, Germany; Cat. #10104159001), 1% bovine serum albumin (BSA), and 1.5 mM CaCl_2_, using gentleMACS (with C Tubes; Miltenyi Biotec, Bergisch Gladbach, Germany; Cat #130-093-235). Tissue homogenates were incubated at 37 °C for 30 min with intermittent mechanical agitation, filtered through 40-µm cell strainers, and centrifuged at 300× *g* for 10 min at 4 °C, and the cell pellet was resuspended in PBS containing 2% fetal bovine serum (FBS). The red blood cells were removed using ammonium-chloride-potassium (ACK) lysis buffer for 1–2 min at room temperature, followed by immediate washing with PBS containing 2% FBS.

### 4.4. EpCAM-Based Cell Depletion and Antibody Staining for Cell Sorting

To enrich for non-epithelial cell populations, EpCAM-positive epithelial cells were depleted from the whole-kidney cell suspension by magnetic-activated cell sorting (MACS) using anti-EpCAM microbeads (Miltenyi Biotec, Bergisch Gladbach, Germany; Cat. #130-061-101) according to the manufacturer’s instructions. Briefly, kidney cell suspensions (see above) were resuspended in a sorting buffer [PBS supplemented with 2% BSA, 2 mM EDTA, and 0.02% sodium azide (NaN_3_)] at a density of 1 × 10^7^ cells per 80 µL buffer. Cells were incubated with 20 µL anti-EpCAM microbeads per 1 × 10^7^ cells for 15 min at 4 °C with gentle mixing. Following incubation, cells were washed with sorting buffer and centrifuged at 300× *g* for 5 min at 4 °C to remove supernatant containing unbound beads. The cell pellet was then resuspended and applied onto LS columns (Miltenyi Biotec, Cat. #130-042-401) pre-equilibrated with sorting buffer and placed in a magnetic separator (Miltenyi Biotec, Cat. #130-092-168). During separation, EpCAM-positive cells labeled with magnetic beads were retained within the column, whereas unlabeled EpCAM-negative cells passed through and were collected as the flow-through fraction. The column was subsequently washed 3 times with sorting buffer to maximize recovery of EpCAM-negative cells. All procedures were performed at 4 °C to preserve cell viability and maintain surface antigen integrity for downstream staining and cell sorting.

For antibody staining, EpCAM-negative cells were resuspended in staining buffer consisting of PBS supplemented with 2% FBS. Cells were incubated for 30 min at 4 °C in the dark with PerCP-conjugated rat anti-mouse CD45 antibody (BioLegend, San Diego, CA, USA; Cat. #103132; 1:50), rabbit anti-Nephrin antibody (Abcam, Cambridge, UK; Cat. #ab136894; 1:100), and APC-conjugated Syrian hamster anti-mouse podoplanin (Pdpn) antibody (BioLegend, Cat. #127410; 1:50). After primary antibody incubation, cells were washed and incubated with Alexa Fluor 488-conjugated goat anti-rabbit IgG secondary antibody (Thermo Fisher Scientific, Waltham, MA, USA; Cat. #A32731; 1:200) against the anti-Nephrin antibody for 30 min at 4 °C in the dark. Cells were then washed twice with staining buffer prior to sorting. To define gating thresholds and control for nonspecific binding, isotype-matched IgG control antibodies were included in parallel staining conditions.

Cell sorting and gating used a BD FACSAria III (BD Biosciences, San Jose, CA, USA) equipped with 488 nm, 561 nm, and 640 nm lasers. Fluorescence signals were detected using the following bandpass filters: FITC/Alexa Fluor 488 (530/30 nm), PE (575/26 nm), PerCP (695/40 nm), and APC (660/20 nm).

The following sequential gating strategy was applied to identify the target population: First, cellular events were selected based on forward scatter area (FSC-A) versus side scatter area (SSC-A) to exclude debris and non-cellular particles. This gate was defined using unstained controls to ensure inclusion of intact cells while excluding low-scatter events. Second, doublets and cell aggregates were excluded by gating on forward scatter height (FSC-H) versus FSC-A, ensuring that only singlet cells were retained for downstream analysis. Third, within the singlet population, hematopoietic cells were excluded based on CD45 expression, detected in the PerCP channel (488 nm excitation/695/40 nm emission). Fourth, podocyte populations were excluded by removing Nephrin-positive (Nephrin^+^) cells, detected in the FITC/Alexa Fluor 488 channel (488 nm excitation/530/30 nm emission). Finally, within the CD45^−^/Nephrin^−^ singlet population, Pdpn-positive (Pdpn^+^) cells were identified in the APC channel (640 nm excitation/660/20 nm emission) and defined as the target lymphatic-like endothelial population. Cell viability gating was not used because, in our experience, such an additional step did not improve yields, but could cause unintended damage to sorted cells. Samples from six animals were pooled for subsequent bulk RNA-Seq analysis.

### 4.5. Pdpn^+^ Cell Culture and RNA Sequencing

Following isolation, EpCAM^−^CD45^−^Nephrin^−^Pdpn^+^ cells were resuspended in endothelial cell medium (ECM; ScienCell, Carlsbad, CA, USA; Cat. #1001) supplemented with 5% FBS, 1% endothelial cell growth supplement (ECGS), and 1% penicillin-streptomycin. Cells were maintained at 37 °C in a humidified incubator with 5% CO_2_ for recovery for ~6 h. At the end of the culture period, dead cells and debris were removed by gentle aspiration, and remaining cells were harvested after rinsing and gentle dissociation, pelleted by centrifugation at 300× *g* for 5 min at 4 °C, and processed for RNA extraction.

Because of the scarcity of the targeted cell population, for RNA sequencing, kidney homogenates from 3 mice were pooled and EpCAM^−^CD45^−^Nephrin^−^Pdpn^+^ cells isolated. Total RNA was extracted using TRIzol reagent (Invitrogen, Carlsbad, CA, USA; Cat. #15596026). RNA concentration and quality were assessed, including spectrophotometric measurements (OD260/280). RNA samples with OD260/280 ratios of 1.8–2.1 were used. Poly(A)-selected RNA was then isolated and used to generate strand-specific sequencing libraries using Illumina-compatible library preparation kits according to the manufacturer’s instructions. Libraries were quantified and assessed for quality prior to submitting to the commercial vendor Genomics BioSci & Tech Co. (New Taipei City, Taiwan) for sequencing. Paired-end sequencing (2 × 150 bp) was performed on a NovaSeq 6000 platform (Illumina, San Diego, CA, USA). To minimize batch effects, libraries were randomized across sequencing lanes. Sequence data have been deposited in the NCBI Gene Expression Omnibus (GEO) under accession number GSE308286 [https://www.ncbi.nlm.nih.gov/geo/query/acc.cgi?acc=GSE308286 (accessed on 26 July 2026)].

### 4.6. In Vitro Cell Culture and Functional Assays

Cell lines were obtained from the American Type Culture Collection (ATCC, Manassas, VA, USA; RRID:CVCL_0307). The representative human ccRCC cell lines from the NCI-60 collection were used for xenografts (see below): 786-O (RRID:CVCL_1051; with homozygous *VHL* mutation and inactivated *p53* and *PTEN* genes) and A498 (RRID:CVCL_1056; with homozygous *VHL* mutation, wild-type *p53* and *PTEN*, and mutated *SETD2*). Both cell lines were transfected with luciferase-expressing plasmids. Non-cancerous human proximal tubule epithelial cells (HK-2) were cultured in Keratinocyte-SFM supplemented with bovine pituitary extract (BPE), epidermal growth factor (EGF), and 1% penicillin-streptomycin at 37 °C in a humidified atmosphere containing 5% CO_2_. The *VHL* KO was generated using the CRISPR/Cas9 gene editing method, as described below. To assess functional responses, *VHL* KO HK-2 cells were treated with recombinant human CXCL13 (50 ng/mL; R&D Systems, Minneapolis, MN, USA; Cat #801-cx-025). Ki67 immunofluorescence staining was conducted to assess proliferation, and the percentage of Ki67^+^ nuclei was quantified using the ImageJ v2 software (https://imagej.net/ij/, accessed on 26 July 2026). Growth curves were analyzed using the CCK8 Assay (Abcam). Scratch-wound-healing assays were used to evaluate two-dimensional (2D) migration in the presence of Mitomycin C (10 μg/mL) to minimize the confounding factor of proliferation, whereas three-dimensional (3D) invasion was assessed using Transwell Boyden chambers (Thermo Fisher Scientific, Waltham, MA, USA; Cat #CLS3422) with Matrigel-coated inserts in minimal media. Recombinant human CXCL13 was added to the lower chamber as described above. The migrated and invaded cells were fixed after 24 h, stained with crystal violet, and counted in five randomly selected fields per membrane. All in vitro experiments were performed in technical triplicates of biological triplicate.

All cultured cells were grown from frozen stocks and propagated for no more than 10 passages to minimize genetic shift and potential cross-contamination by other cells. Passaged cells were not re-frozen or re-stocked. Cell cultures were routinely checked for mycoplasma contamination using an EZ-PCR Mycoplasma Detection Kit (Sartorius, Göttingen, Germany; Cat #20-700-20) Details of the media and supplements are shown in Table S1, and all the antibodies used are shown in Table S2.

### 4.7. CRISPR/Cas9-Mediated Knockout of VHL in HK-2 Cells

*VHL*-KO HK-2 cells were generated using a lentiviral CRISPR/Cas9 system as previously described with modifications [48]. Briefly, complementary oligonucleotides encoding a *VHL*-targeting single-guide RNA sequence were annealed and cloned into the lentiCRISPRv2 vector (Addgene, Watertown, MA, USA; Cat. #52961), which expresses both SpCas9 and a puromycin-resistance cassette. The *VHL*-targeting oligonucleotide sequences were obtained from the GenScript gRNA 2016 database: [https://www.genscript.com/gRNA-database.html (accessed on 26 July 2026)] as follows: sgVHL2 (VHLKO2): 5′-GTGACTAGGCTCCGGACAACC-3′; and sgVHL3 (VHLKO3): 5′-GCAGGTCGCTCTACGAAGATC-3′. The resulting lentiCRISPRv2-sgVHL construct was used for lentiviral production together with the lentiviral packaging plasmid psPAX2 (Addgene, Cat. #12260) and pMD2.G (Addgene, Cat. #12259). Empty lentiCRISPRv2 vector was used as the control. Lentiviral particles were produced in packaging cells and subsequently used to transduce HK-2 cells. After transduction, cells were selected with puromycin at 1 µg/mL for 7 days to establish pooled stable *VHL*-KO HK-2 cell populations and stored. Each thawed fraction was regrown in the presence of antibiotic, and the antibiotic was removed before experimentation. The regrown cells were never re-stocked. *VHL* KO efficiency was confirmed by Western blotting before subsequent experiments (Figure S1). *VHL* KO using the sgVHL2 guide RNA was used throughout this study.

### 4.8. Quantitative Reverse Transcription-PCR (qRT-PCR)

Total RNA was extracted using TRIzol reagent (Invitrogen, Cat. #15596026) following the manufacturer’s instructions. RNA concentration and purity were assessed by spectrophotometry (NanoDrop Ultra, Thermo Fisher Scientific, Waltham, MA, USA; Cat #NDULTRAGL), and only samples with an A260/A280 ratio between 1.8 and 2.1 were considered acceptable. RNA integrity was further evaluated using a Bioanalyzer system (Agilent Technologies), which provides electropherogram profiles and calculates the RNA integrity number (RIN). Only RNA samples with a RIN ≥ 7.5 were used for qRT-PCR.

cDNA was synthesized from DNase-treated total RNA using the High-Capacity cDNA Reverse Transcription Kit (Applied Biosystems, Waltham, MA, USA; Cat. #4368814) according to the manufacturer’s instructions. Briefly, 500 ng of total RNA was used as input for each reaction and reverse transcribed in a 20 µL reaction containing reverse transcription buffer, dNTP mix, random primers, MultiScribe™ reverse transcriptase, and RNase inhibitor. Prior to reverse transcription, RNA samples were briefly denatured at 95 °C and kept on ice to reduce secondary structure formation. The reverse transcription reaction was performed under the following thermal conditions: 25 °C for 10 min (primer annealing), 37 °C for 120 min (cDNA synthesis), and 85 °C for 5 min (enzyme inactivation). The cDNA samples were then diluted with nuclease-free water (1:5) and stored at −80 °C until use.

Quantitative real-time PCR (qPCR) was performed using iTaq Universal SYBR Green Supermix (Bio-Rad, Hercules, CA, USA; Cat. #1725121) on a Bio-Rad CFX Real-Time PCR System, and fluorescence data were acquired and analyzed using the CFX Maestro v2.3 software. Gene-specific primers are listed in Supplementary Table S3. The primer sequences were based on previously validated primer sequences available from OriGene [www.origene.com/catalogsearch/result/index/?oti_sub_category=18067&q=primer+ (accessed on 26 July 2026)] and were sourced from Genomics BioSci & Tech Co. (New Taipei City, Taiwan). Each reaction was prepared in a final volume of 20 µL containing 10 µL of 2 × SYBR Green Supermix, 1 µL of forward primer, and 1 µL of reverse primer (10 µM stock solutions; final concentration 0.5 µM each, corresponding to ~10 pmols per primer per reaction), 2 µL of cDNA template (i.e., equivalent of ~10 ng of the original RNA used in cDNA synthesis), and 6 µL of nuclease-free water. All reactions were performed in technical triplicate. No-template controls were included to monitor contamination, and no-reverse transcriptase controls were used to confirm the absence of genomic DNA contamination. PCR amplification was carried out with an initial denaturation at 95 °C for 3 min, followed by 40 cycles of denaturation at 95 °C for 10 s and annealing/extension at 60 °C for 30 s. A melt curve analysis was performed at the end of each run to confirm amplification specificity, and only reactions displaying a single, specific melt peak and a clear exponential amplification curve were included in downstream analysis.

Cq (quantification cycle) values were determined using CFX Maestro software (Bio-Rad), in which Cq represents the cycle number at which the fluorescence signal exceeds a defined threshold within the exponential phase of amplification. Threshold and baseline settings were initially determined automatically by the software and verified manually to ensure consistency across samples. A lower Cq value indicates higher transcript abundance. PCR amplification was performed for 40 cycles. Cq < 35 was considered indicative of robust and reliable target detection; Cq values between 35 and 38 were interpreted as low to very low expression, and Cq of 39–40 or no threshold crossing was considered below the detection limit of the assay. Reactions exhibiting nonspecific amplification, irregular amplification curves, or high variability among technical replicates were excluded from further analysis. To account for variation in RNA inputs and reverse transcription efficiency, gene expression levels were normalized to the housekeeping gene *ACTB* (*β-Actin*). Note that the often-used reference gene *GAPDH* is not suitable for *VHL*-deficient cells since *GAPDH* is a HIF target [49]. For each sample, the mean Cq value from technical triplicates was used to calculate ΔCq values as follows: ΔCq = Cq(target gene) − Cq(ACTB).

### 4.9. Human ccRCC Tissue

Archived human ccRCC tissues were collected from patients at National Taiwan University Hospital (NTUH), with approval from the Internal Review Board of NTUH (IRB no. 202403140RINA; date: 27 May 2024). Tissue arrays containing paraffin sections of human kidney cancer tissue were purchased from US Biomax Inc. (Cat. #KD241; Derwood, MD, USA).

### 4.10. Histology and Immunostaining

The kidneys were harvested and fixed in 4% paraformaldehyde at 4 °C overnight. The tissues were then processed for paraffin embedding. The embedded samples were sectioned at 3 μm and stained with hematoxylin and eosin (H&E) for histopathological evaluation. Paraffin-embedded samples were analyzed using immunohistochemistry (IHC) and immunofluorescence (IF). Paraffin sections were de-paraffinized, rehydrated, and retrieved as previously described [31]. Briefly, the tissue sections were subjected to antigen retrieval in citrate buffer (pH 6.0) and heated to just boiling in a microwave oven. Sections were then blocked with 5% normal serum and incubated with primary antibodies. Detection was achieved using species-specific secondary antibodies conjugated to fluorophores (Alexa Fluor 488, 586, or 647) or horseradish peroxidase (HRP). Nuclear staining was performed with DAPI (4′,6-diamidino-2-phenylindole). Antibody usage is shown in Table S2. Imaging conditions were standardized across experimental groups to ensure comparability. Quantitative analysis of marker expression and positive cell counts was conducted using ImageJ [https://imagej.net/ij/ (accessed on 26 July 2026)]; RRID:SCR_003070)] or CellProfiler v4.2.8 [https://cellprofiler.org (accessed on 26 July 2026); RRID: SCR_007358] software.

### 4.11. Lymphatic Drainage Assay

To evaluate renal lymphatic function, 10 µL of 1% Evans Blue Dye (EBD; Sigma-Aldrich, Burlington, MA, USA; Cat #E2129) in PBS was injected into the subcapsular space of the kidney. After 30 min, the mice were euthanized, and their kidneys excised, rinsed, blotted dry, and weighed. Each kidney was sliced and incubated in 1 mL of 100% formamide at 60 °C for 24 h to extract the retained dye. The optical density (OD) of the supernatant was measured at 620 nm using a spectrophotometer, and the EBD content was normalized to the tissue weight. Increased dye retention is interpreted as an indicator of impaired lymphatic drainage.

### 4.12. Western Blot

Cells or tissue samples were lysed in RIPA buffer containing a protease and phosphatase inhibitor cocktail. The lysates were incubated on ice for 30 min and then clarified by centrifugation at 14,000× *g* for 15 min at 4 °C, and protein concentrations were measured using the BCA Protein Assay Kit (Thermo Fisher Scientific, Waltham, MA, USA; Cat. #23225). Equal amounts of proteins (50 μg) were separated by SDS-PAGE on 8–12% polyacrylamide gels and transferred to PVDF membranes. The membranes were blocked with 5% non-fat dry milk or BSA in TBST (Tris-buffered saline with 0.1% Tween-20) for 1 h at room temperature, followed by overnight incubation at 4 °C with primary antibodies. After washing, the membranes were incubated with appropriate HRP-conjugated secondary antibodies for 1 h at room temperature. Protein bands were visualized using enhanced chemiluminescence (ECL; Thermo Fisher Scientific, Waltham, MA, USA; Cat. #32106) reagents and imaged using the ChemiDoc XRS+ System (Bio-Rad). Band intensities were quantified using Image Lab software v5.2.1 (RRID:SCR_014210) and normalized to the corresponding loading controls. Details of the reagents are listed in Table S1 and primary and secondary antibodies in Table S2.

### 4.13. ALK1/2 Inhibitor Treatment

LDN-212854 (Selleckchem, Houston, TX, USA; Cat. #S7147) is a specific ALK1/2 inhibitor. The treatment regimen for mice follows the published report [50] and the supplier’s instructions. Briefly, 20 μL of the 25 mg/mL DMSO stock solution was diluted 50× into a solution consisting of 0.5 mg/mL LDN-212854, 40% PEG300, 20% Tween-80, and 2% DMSO. The inhibitor was used at 9 mg/kg/dose via IP injection.

### 4.14. Anti-BMP10 Antibody Treatment

BMP10 neutralization in the VhlhKO mouse model was performed using a mouse monoclonal anti-human/mouse BMP10 antibody (clone 462732, mouse IgG2A; R&D Systems, Minneapolis, MN, USA; Cat. #MAB2926). The antibody was reconstituted in sterile 1× PBS at 0.5 mg/mL as recommended by the manufacturer and stored under sterile conditions until use. For in vivo treatment, Ctrl and VhlhKO mice received anti-BMP10 antibody at 5 mg/kg by intravenous tail-vein injection once per week for 4 consecutive weeks. The control group received an equivalent amount of isotype-matched control IgG (Mouse IgG2A Isotype Control, R&D Systems, Cat. #MAB003). Mice were monitored throughout the treatment period for changes in health conditions and body weight.

### 4.15. BMP10 Neutralization Assay in Xenograft Mouse Model

786-O cells expressing luciferase (786-O^Luc^) or A498 cells expressing luciferase (A498^Luc^) were grown in RPMI-1640 supplemented with 10% FBS and 1% Pen-Strep until ~60% confluency, then 5 × 10^5^ cells were suspended in 10 μL 1× PBS containing 50% Matrigel Growth Factor Reduced (Corning, Glendale, AZ, USA; Cat. #356238). The mixture was then injected into the subrenal capsular space of the left kidney of immunodeficient mice (NOD.CB17-Prkdc^scid^/NcrCrl) using a 33G Hamilton syringe while the mice were anesthetized with isoflurane. Starting on Day 7 post-implantation, mice were randomized into two groups (*n* = 12 per group). The control group received isotype control IgG antibody via tail vein injection, whereas the treatment group received an anti-BMP10 neutralizing antibody (5 mg/kg weight; BioLegend, San Diego, CA, USA; Cat. #618104) once a week for 4 weeks.

The growth and metastasis of cancer cells were monitored weekly by luminescence signal using the IVIS Lumina LT Series III system (Revvity, Waltham, MA, USA). At the end of the experiment (Day 35), tissues were harvested and embedded in paraffin blocks for analysis. Cell viability prior to implantation was confirmed using Trypan Blue (Thermo Fisher Scientific, Waltham, MA, USA; Cat #15250061) exclusion, ensuring over 90% viability, and all injections into the kidney were performed within one hour of cell preparation. Post-operative care included administration of analgesics and daily monitoring, with all procedures adhering to a protocol pre-approved by the Institutional Animal Care and Use Committee of China Medical University (CMUIACUC-2024-034).

### 4.16. Statistical Analysis

Comparisons between two groups were performed using an unpaired two-tailed Student’s *t*-test. For comparisons involving more than two groups, one-way analysis of variance (ANOVA) was conducted, followed by Tukey’s multiple comparisons post hoc test. *p*-values were interpreted as statistically significant at *p* < 0.05. All statistical analyses were performed using GraphPad Prism 11.0.2 (GraphPad Software, San Diego, CA, USA; RRID:SCR_002798) or the R statistical software 4.3.2 (RRID:SCR_001905). The sample sizes (*n*) are reported in the corresponding figure legends.

## 5. Conclusions

The lymphatic system as a conduit for immune and metastatic cells can serve both pro- and anti-tumor functions. It is, therefore, generally not considered a good candidate for therapeutic purposes, although theoretically it should be an accessible drug target. In this study, we identified a highly specific reciprocal signaling mechanism between ccRCC tumor cells and a unique group of tumor-associated dysLECs that promote ccRCC progression and metastasis. This signaling mechanism is initiated by BMP10 specifically produced by tumor cells, and targeting this signaling mechanism represents a promising new therapeutic strategy against ccRCC.

## Data Availability

The original contributions presented in this study are included in the article/Supplementary Material. Further inquiries can be directed to the corresponding author(s). Sequence data have been deposited in the NCBI Gene Expression Omnibus (GEO) under accession number GSE308286 [https://www.ncbi.nlm.nih.gov/geo/query/acc.cgi?acc=GSE308286 (accessed on 26 July 2026)].

## Supplementary Materials

The following supporting information can be downloaded at: https://www.mdpi.com/article/10.3390/ijms27156994/s1.
