# Supplementary material for: Tumor-Induced PDPN+ Lymphatic-like Endothelial Cells Promote Clear-Cell Renal Cell Carcinoma Progression Through Reciprocal BMP10-CXCL13 Signaling
Source: Int J Mol Sci. 2026 Aug 4;27(15):6994. doi: 10.3390/ijms27156994 (PMC13467439; doi:10.3390/ijms27156994)
Supplement: Supplementary file 1 [file ijms-27-06994-s001.zip › Supplementary Figures.pdf]

Supplementary Figures

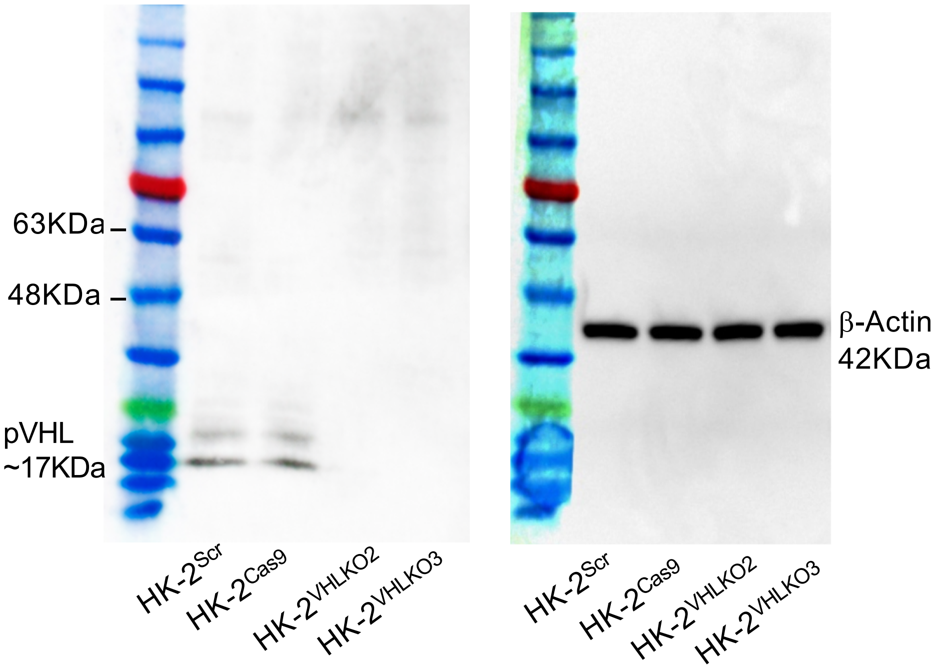

Figure S1. Validation of *VHL* inactivation in HK-2 cells. HK-2 cells were transfected with vector (puromycin resistance) expressing Cas9 only (HK-2^{Cas9}) or vector expressing Cas9 and *VHL-specific* guide RNA (HK-2^{VHLKO2} or HK-2^{VHLKO3}; see Materials and methods). Antibiotic-resistant cells (Puromycin-resistant) were pooled and stored. Two independent lines from two different guide RNAs (VHLKO2 and VHLKO3) and one control line (Cas9) were maintained. Protein extracts from these lines were Western-blotted for human VHL protein (pVHL) and for the loading control β-Actin. An independent HK-2 cells transfected with vector expressing shRNA of scrambled sequence served as an additional control of un-altered VHL protein expression level. The result shows efficient knockout of the *VHL* gene. The VHLKO2 line was used throughout this study (designated HK-2^{VHLKO}.

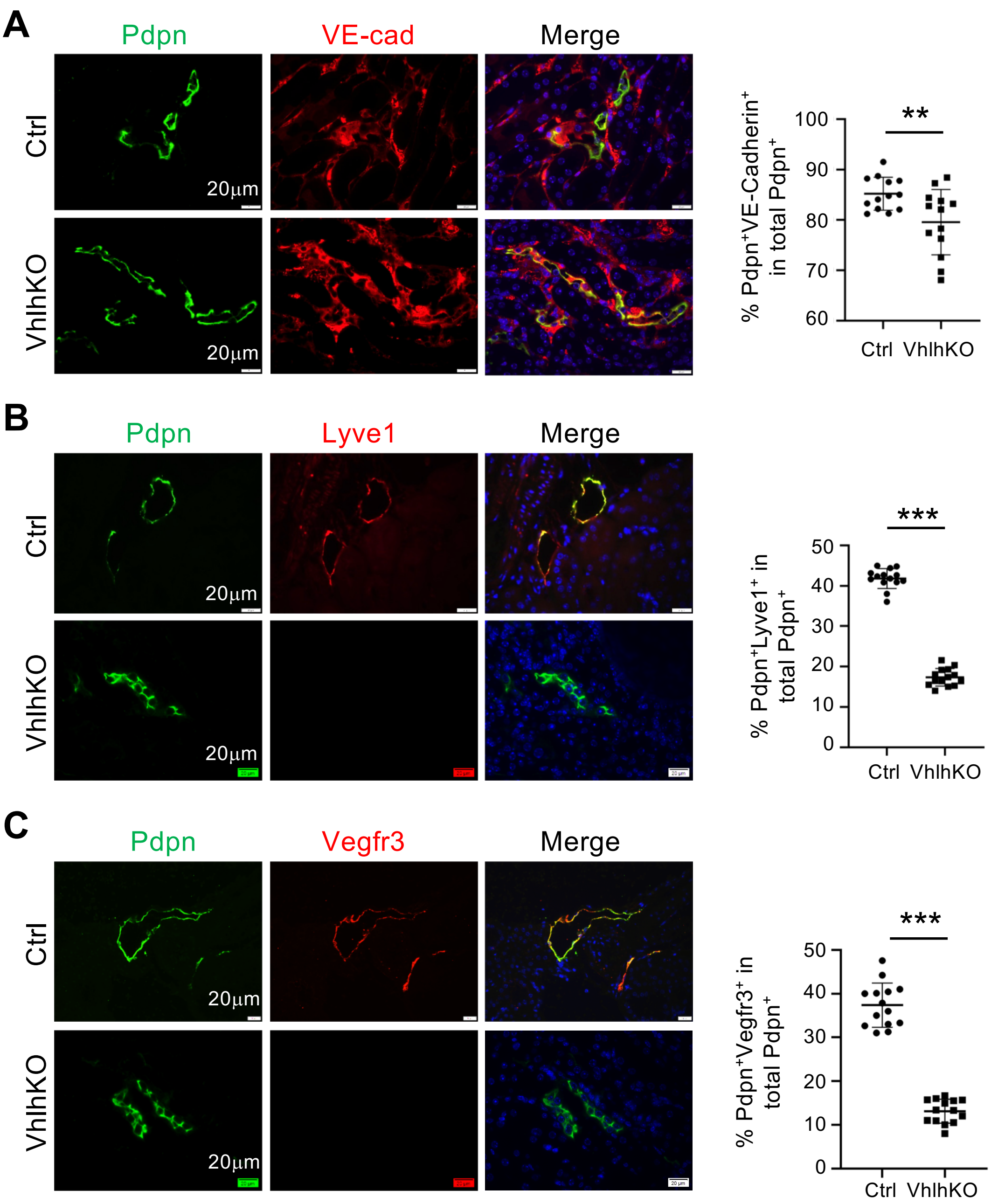

Figure S2. A majority of Pdpn+ lymphatic-like cells in VhlhKO kidney do not express a full set of canonical lymphatic endothelial cell markers. **A,** Kidney sections from Control (Ctrl) and VhlhKO mice were stained for Pdpn and VE-cadherin. About 85% of Pdpn+ cells also express VE-cadherin in Ctrl, and the percentage is reduced in VhlhKO. Scale bars are 20 μm. n=13. **, *P*<0.01. Each data point represents one independent sample. **B,** Kidney sections from Ctrl and VhlhKO mice were stained for Pdpn and Lyve1. About 40% of Pdpn+ cells also express Lyve1 in Ctrl, and the percentage is significantly reduced in VhlhKO to ~15%. Scale bars are 20 μm. n=14. ***, *P*<0.001. Each data point represents one independent sample. **C,** Kidney sections from WT and VhlhKO mice were stained for Pdpn and Vegfr3. About 40% of Pdpn+ cells also express Vegfr3 in Ctrl and the percentage is reduced in VhlhKO to less than 15%. Scale bars are 20 μm. n=14. ***, *P*<0.001. Each data point represents one independent sample.

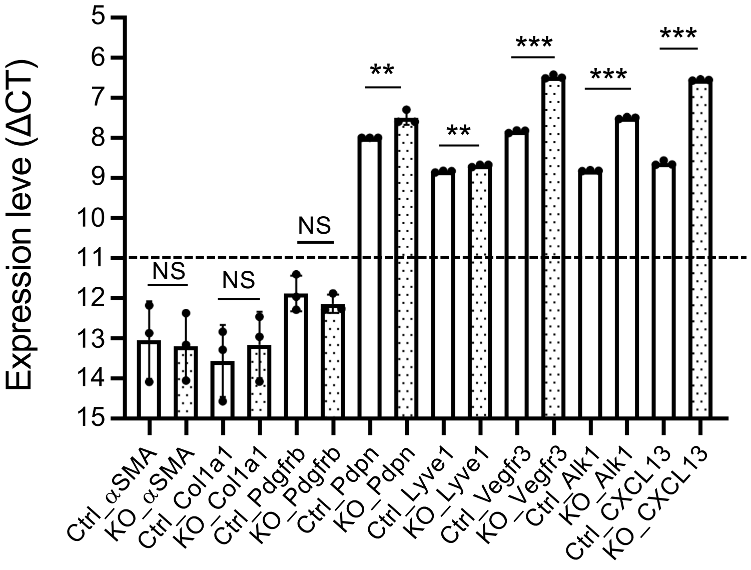

Figure S3. The EpCAM⁻CD45⁻Nephrin⁻Pdpn⁺ cells are not fibroblastic cells. EpCAM⁻CD45⁻Nephrin⁻Pdpn⁺ cells were isolated from *Hox-b7-Cre-GFP/+* (Ctrl) or *Hox-b7-Cre-GFP/+; Vhlh$^{loxP/loxP}$* (KO) mouse kidneys. Expression levels of *αSMA, Col1a1, Pdgfrb, Pdpn, Lyve1, Vegfr3/Flt4, Alk1,* and *Cxcl13* were quantified by quantitative reverse transcription-PCR (qRT-PCR). Raw cycle threshold (Ct) values were normalized to an internal reference gene (*β-Actin*) to generate ΔCt values (ΔCt = Ct_target−Ct_reference). The relative expression level is presented as ΔCt, such that higher values indicate lower gene expression. The reference gene *β-Actin* shows a Ct of ~24. Since Ct values greater than 35 are considered low to no expression, the ΔCT of 11 is the threshold. ΔCT lower than 11 indicates clear and reliable target detection. Each data point represents a biological replicate, and bars indicate mean ± SD (n=3). Statistical significance between Ctrl and KO groups was determined using unpaired two-tailed Student's *t*-test. ns, no significance; **, *P*<0.01; ***, *P*<0.001. The raw data of Ct and ΔCt are shown in Supplementary Data 1.

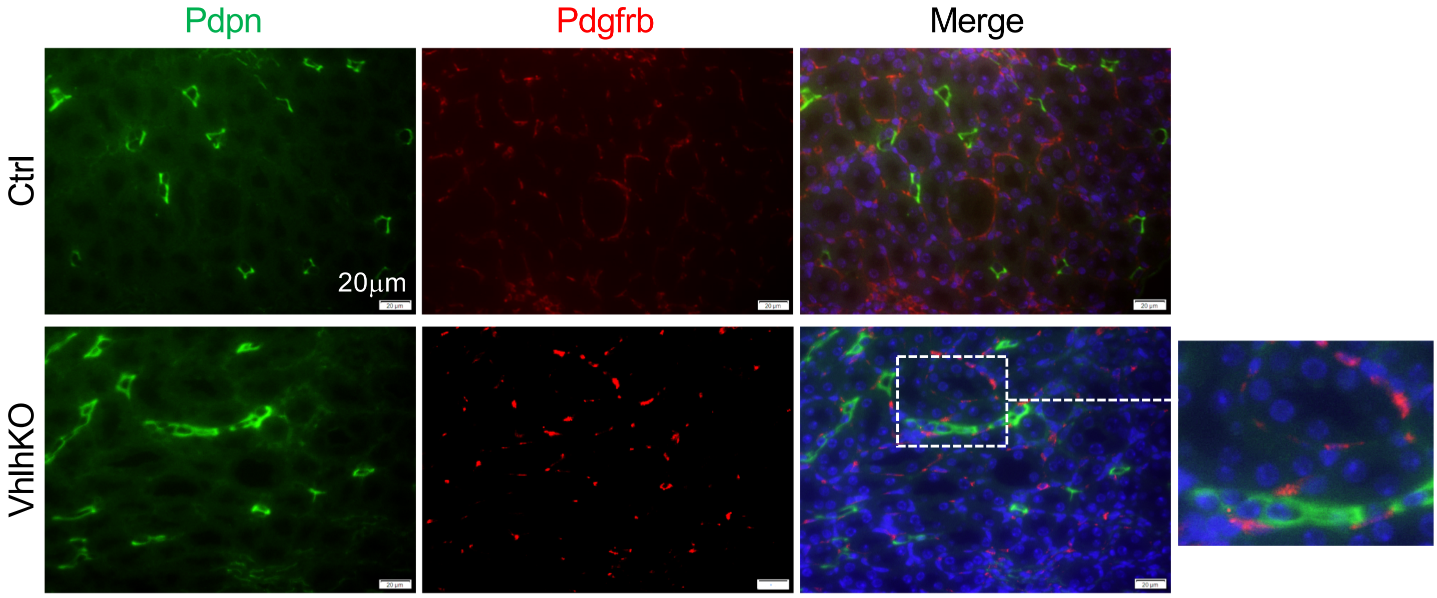

Figure S4. Pdpn⁺ cells do not co-localize with the pericyte marker Pdgfrb in VhlhKO kidneys. Representative double immunofluorescence staining of kidney sections for Pdpn (green) and Pdgfrb (red) in Ctrl and VhlhKO mice. Nuclei were counterstained with DAPI (blue). There are few $Pdgfrb^+$ cells in Ctrl. In contrast, $Pdgfrb^+$ cells are numerous in VhlhKO. These $Pdgfrb^+$ cells can be seen juxtaposing $Pdpn^+$ cells or separate from the $Pdpn^+$ structures but do not overlap, indicating that $Pdpn^+$ dysLECs are distinct from canonical pericytes or myofibroblasts. Scale bars, 20 μm.

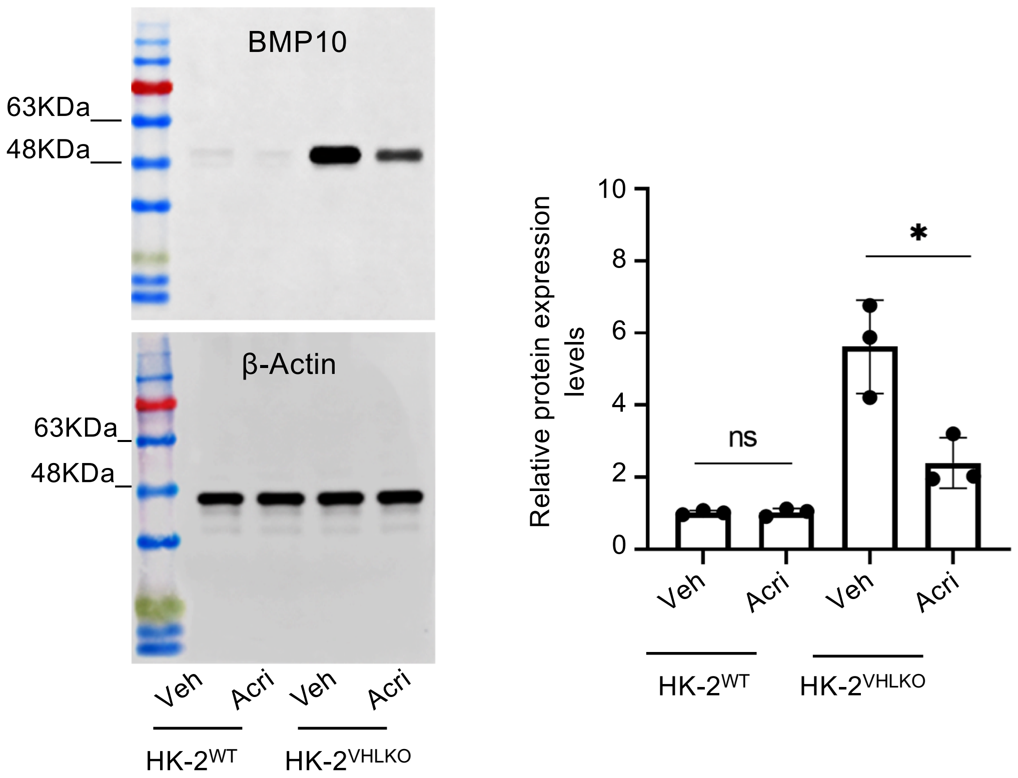

Figure S5. BMP10 expression is increased in HK-2 *VHL*KO cells and is partially attenuated following pharmacologic inhibition of HIF signaling. HK-2 wild-type (WT) and *VHL*-knockout (VHLKO) cells were treated with vehicle control (Veh; DMSO) or the HIF dimerization inhibitor Acriflavine hydrochloride (Acri) at 2.5 μM for 24 hr prior to protein extraction and Western blot analysis. Representative immunoblotting demonstrated no detectable basal BMP10 protein expression in HK-2^{WT} cells with or without Acriflavin treatment, whereas BMP10 expression was markedly elevated in HK2^{VHLKO} cells. Treatment with Acriflavine resulted in reduction of BMP10 protein levels in *VHL*KO cells, suggesting that HIF contributes, at least in part, to BMP10 upregulation following *VHL* loss. HK-2^{WT} and HK2^{VHLKO} cells were cultured in Keratinocyte-SFM supplemented with Bovine Pituitary Extract (BPE), Epidermal Growth Factor (EGF), and 1% penicillin–streptomycin at 37°C in a humidified incubator with 5% CO_2. For HIF inhibition experiments, cells were seeded in 6-well plates and allowed to reach approximately 80% confluence prior to treatment. Acriflavine hydrochloride (MedChemExpress, Cat. No. HY-B0949) was dissolved in dimethyl sulfoxide (DMSO) to prepare a stock solution and diluted in complete culture medium immediately before use. HK-2 WT and VHLKO cells were treated with Acriflavine hydrochloride at various 2.5 μM for 24 hr. Following treatment, cells were harvested for protein extraction and Western blot analysis. Immunoreactive bands were detected using enhanced chemiluminescence (ECL) substrate and visualized with the ChemiDoc XRS+ System (Bio-Rad). β-Actin served as the internal loading control. Each dot represents an individual experiment. n=3, and bars indicate mean ±SD. Statistic significance was determined using an unpaired two-tailed Student's *t*-test. *, $P<0.05$.

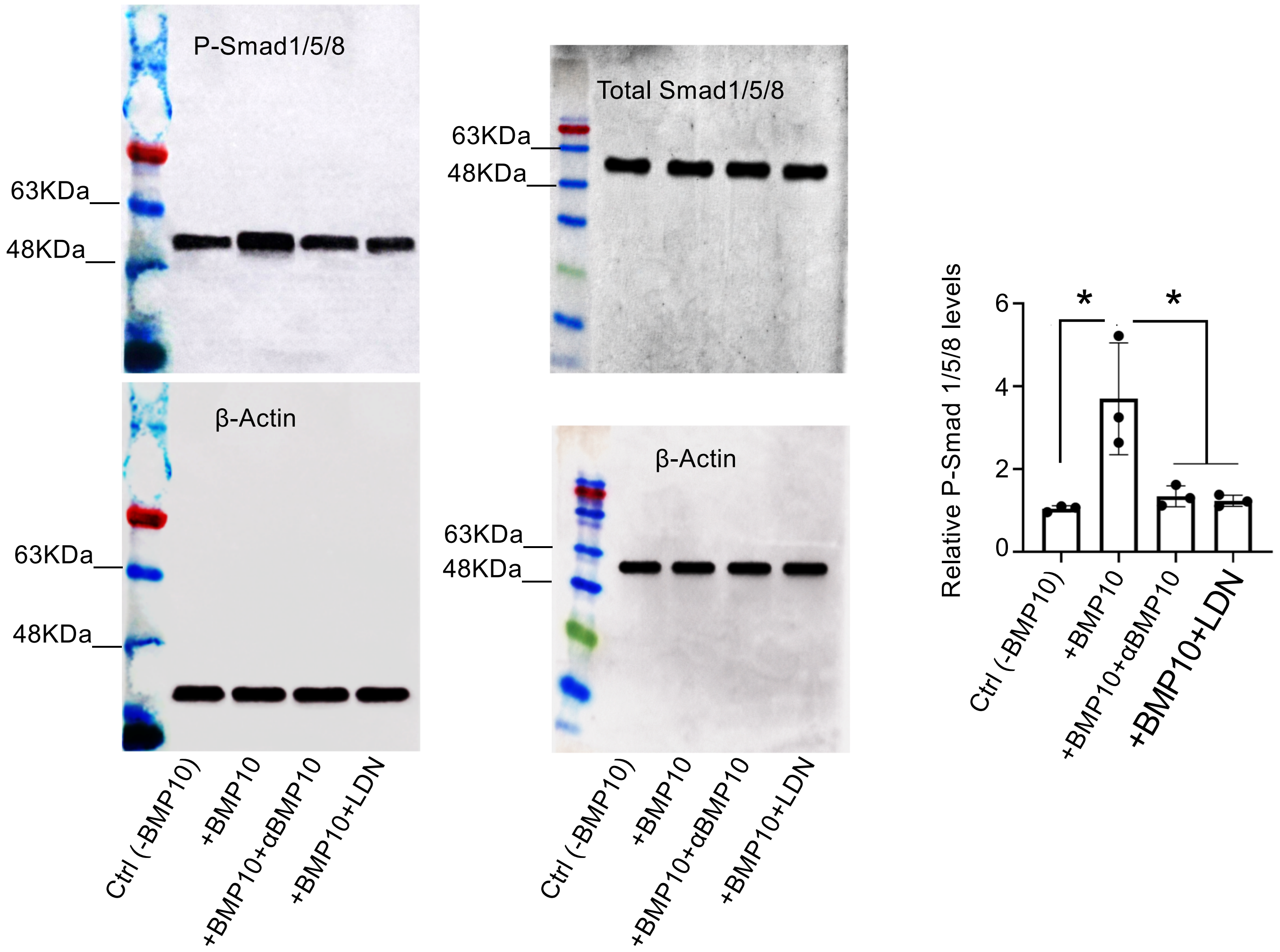

Figure S6. BMP10 induces activation (phosphorylation) of Smad1/5/8. EpCAM$^-$CD45$^-$ Nephrin$^-$Pdpn$^+$ cells were isolated from mouse kidneys as described in Materials and methods. Sorted cells were maintained in Endothelial Cell Medium supplemented with 5% fetal bovine serum (FBS), 1% endothelial cell growth supplement (ECGS), and 1% penicillin-streptomycin solution. Cells were cultured at 37°C in a humidified incubator containing 5% CO_2. After overnight attachment, cells were serum-starved in low-serum ECM containing 0.5% FBS for 12 hr prior to stimulation by BMP10. Recombinant mouse BMP10 protein was reconstituted according to the manufacturer's instructions and diluted in culture medium immediately before use. Cells were untreated (Ctrl -BMP10) or treated with 50 ng/ml of recombinant mouse BMP10 for 12 hr. Separate treatment groups were in addition treated with anti-BMP10 neutralizing antibody (αBMP10) at 1 µg/ml, or ALK1/2 inhibitor (LDN-212854) at 2 µM. Cells were harvested for protein extraction and Western blot analysis. Quantification is shown on the right. BMP10 could induce Smad1/5/8 phosphorylation, and both αBMP10 and ALK1/2 inhibitor could inhibit the activation. Each dot represents an individual mouse. n=3, and bars indicate mean ±SD. Statistic significance was determined using an unpaired two-tailed Student's *t*-test. *, P<0.05.

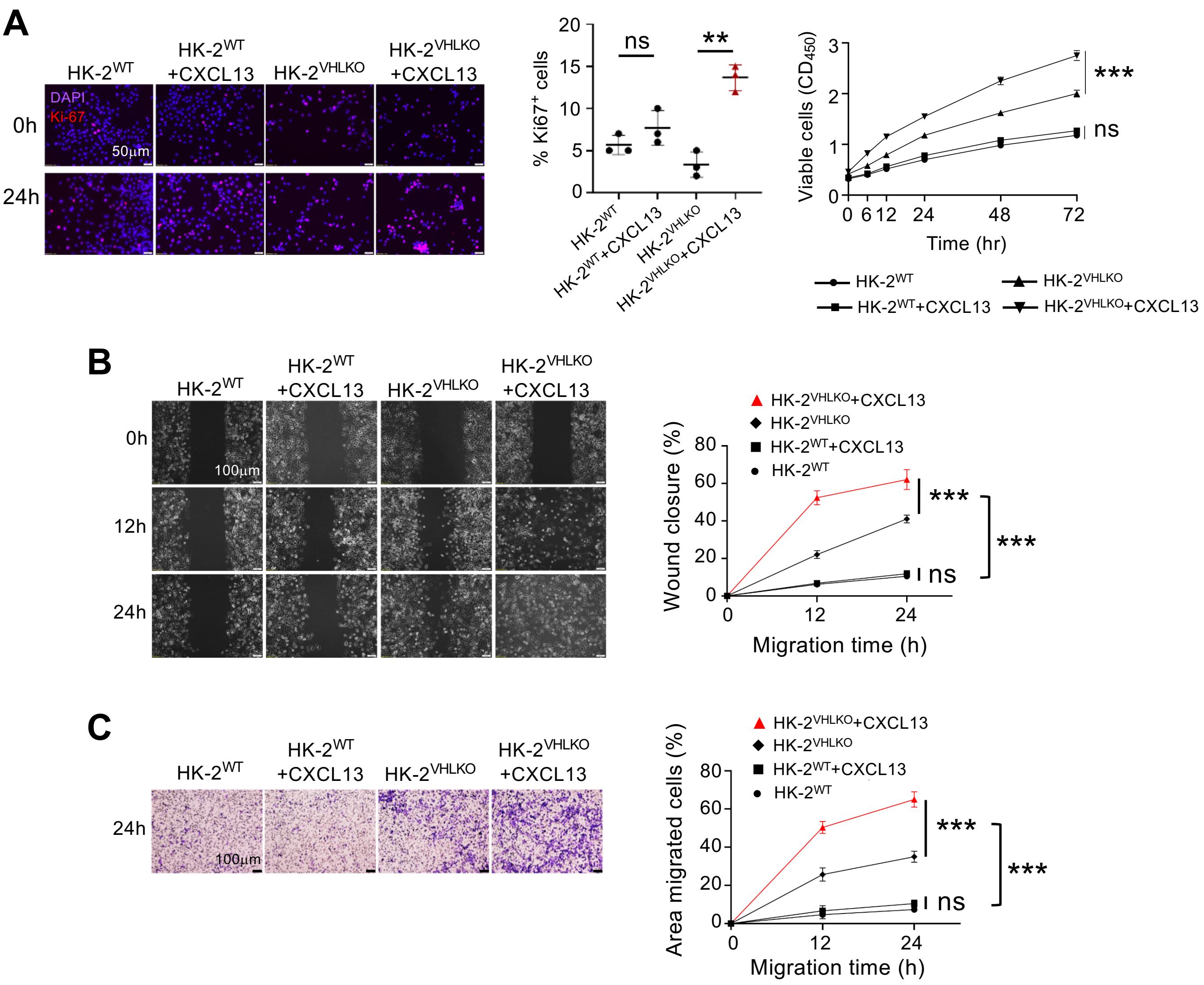

Figure S7. CXCL13 induces proliferation and motility in *VHL*-deficient kidney tubule cells. **A,** Immortalized human non-cancerous kidney tubule cells HK-2 with or without *VHL* knockout (VHLKO or WT, respectively) were grown to ~60% confluency and then treated or untreated with recombinant human CXCL13 (50 ng/ml). 24 hr later cells were stained for Ki67 and quantified. Growth curve analysis (right panel) was also conducted using the CCK-8 assay (Abcam). CXCL13 has no effect on HK-2^{WT} but significantly increases proliferation of HK-2^{VHLKO}. Scale bars are 50 µm. n=3. ns, no significance; **, *P*<0.01. Each data point represents one independent sample. **B,** HK-2^{WT} and HK-2^{VHLKO} were grown to ~80-90% confluency and treated with 10 µg/ml Mitomycin-C. A scratch across the monolayer was produced using a 0.2-ml pipet tip and the cells were treated with CXCL13 as above. At indicated time points, the gap (wound) was measured. CXCL13 has no effect on the migration capacity HK-2^{WT} but significantly increases migration of HK-2^{VHLKO}. Scale bars are 100 µm. n=3. ns, no significance; ***, *P*<0.001. Each data point represents one independent sample. **C,** HK-2^{WT} and HK-2^{VHLKO} were plated on the upper chamber of a Boyden chamber coated with a thin layer of Matrigel to ~60% confluency. The insert was rinsed and placed over the lower chamber with serum-free media. CXCL13 was added to the lower chamber as above. 24 hr later the upper chamber was removed and rinsed. Unmigrated cells were scraped with a cotton swab, and the migrated cells stained with crystal violet. CXCL13 has no effect on the invasive capacity HK-2^{WT} but significantly increases invasion and migration of HK-2^{VHLKO}. Scale bars are 100 µm. n=3. ns, no significance; ***, *P*<0.001. Each data point represents one independent sample. Quantification was performed using the ImageJ software.

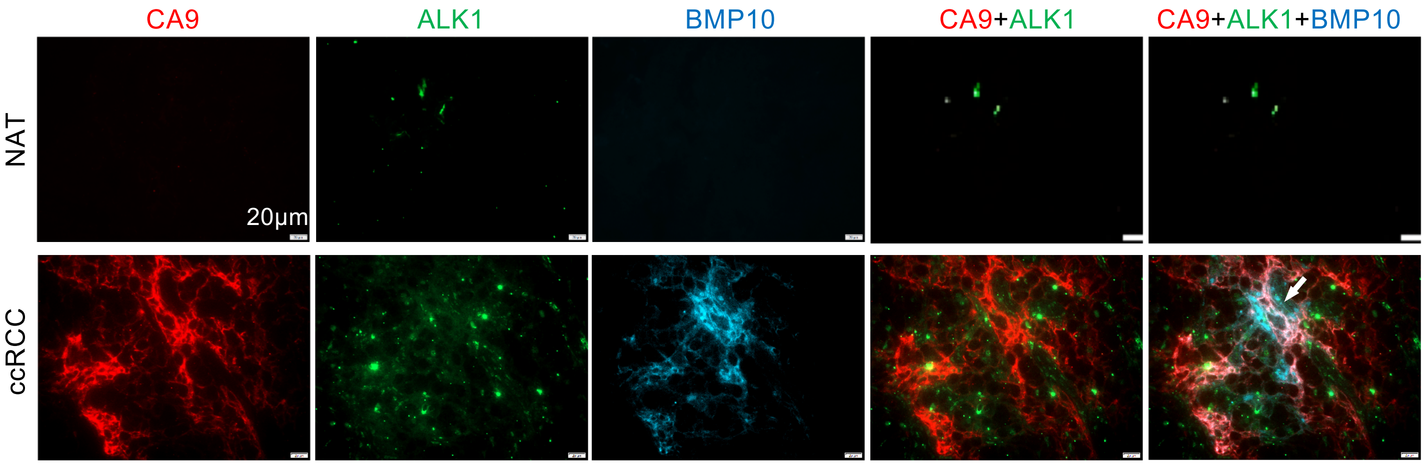

Figure S8. Spatial distribution of CA9, BMP10 and ALK1 in human ccRCC tissues. Representative immunofluorescence images of normal adjacent tissue (NAT) and human clear-cell renal cell carcinoma (ccRCC) sections stained for CA9 (tumor epithelial marker), ALK1, and BMP10. NAT sections show low Alk1 expression, and no detectable CA9 and BMP10 signals. In ccRCC tissue, CA9 marks tumor epithelial cells. BMP10 expression is predominantly localized within $CA9^{+}$ tumor epithelial cells (arrow), whereas ALK1 signal is largely detected in stromal compartments and shows minimal overlap with $CA9^{+}$ tumor cells.

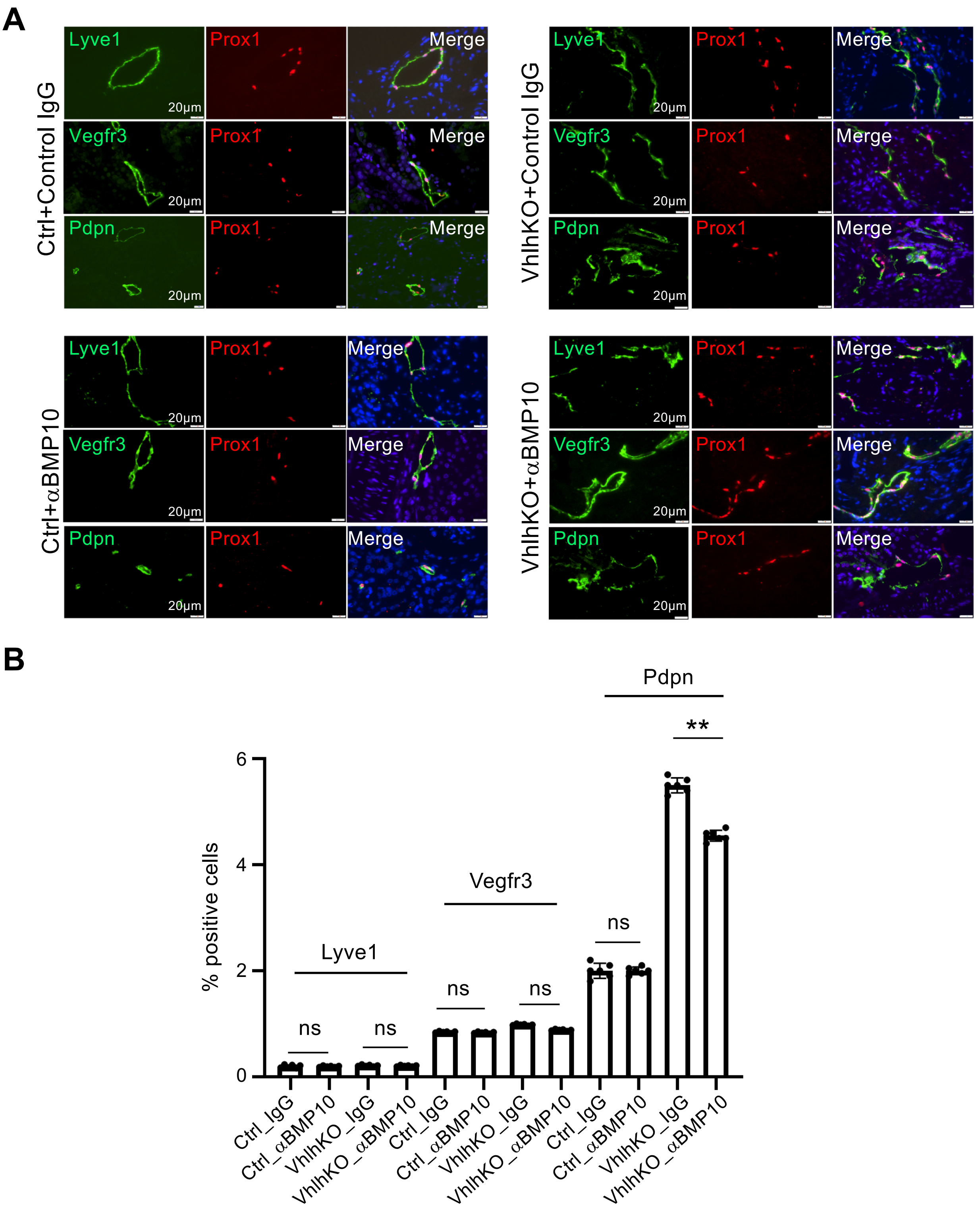

Figure S9. BMP10 is important for development of Pdpn⁺ cells in VhlhKO kidney. **A,** Kidney sections from Ctrl and VhlhKO mice were treated with control IgG or anti-BMP10 neutralizing antibody (αBMP10), and analyzed by immunofluorescence staining for lymphatic markers Lyve1, Vegfr3, and Pdpn (green), together with Prox1 (red) as a lymphatic endothelial cell marker. Nuclei were counterstained with DAPI (blue). Representative images are shown. n=5. Scale bars, 20 µm. **B,** Quantification of Lyve1⁺, Vegfr3⁺, and Pdpn⁺ cells in kidney sections from Ctrl and VhlhKO mice treated with control IgG or anti-BMP10 neutralizing antibody. Positive staining was quantified by CellProfiler software and expressed as percentages of total kidney cells. While Lyve1⁺ and Vegfr3⁺ cell numbers are not affected by BMP10 inhibition, in either Ctrl or VhlhKO, the number of Pdpn⁺ cells in VhlhKO is reduced by anti-BMP10 treatment. Each data point represents an individual mouse. n=5. Error bars, mean ± SD. ns, no significance; **, *P*<0.01.

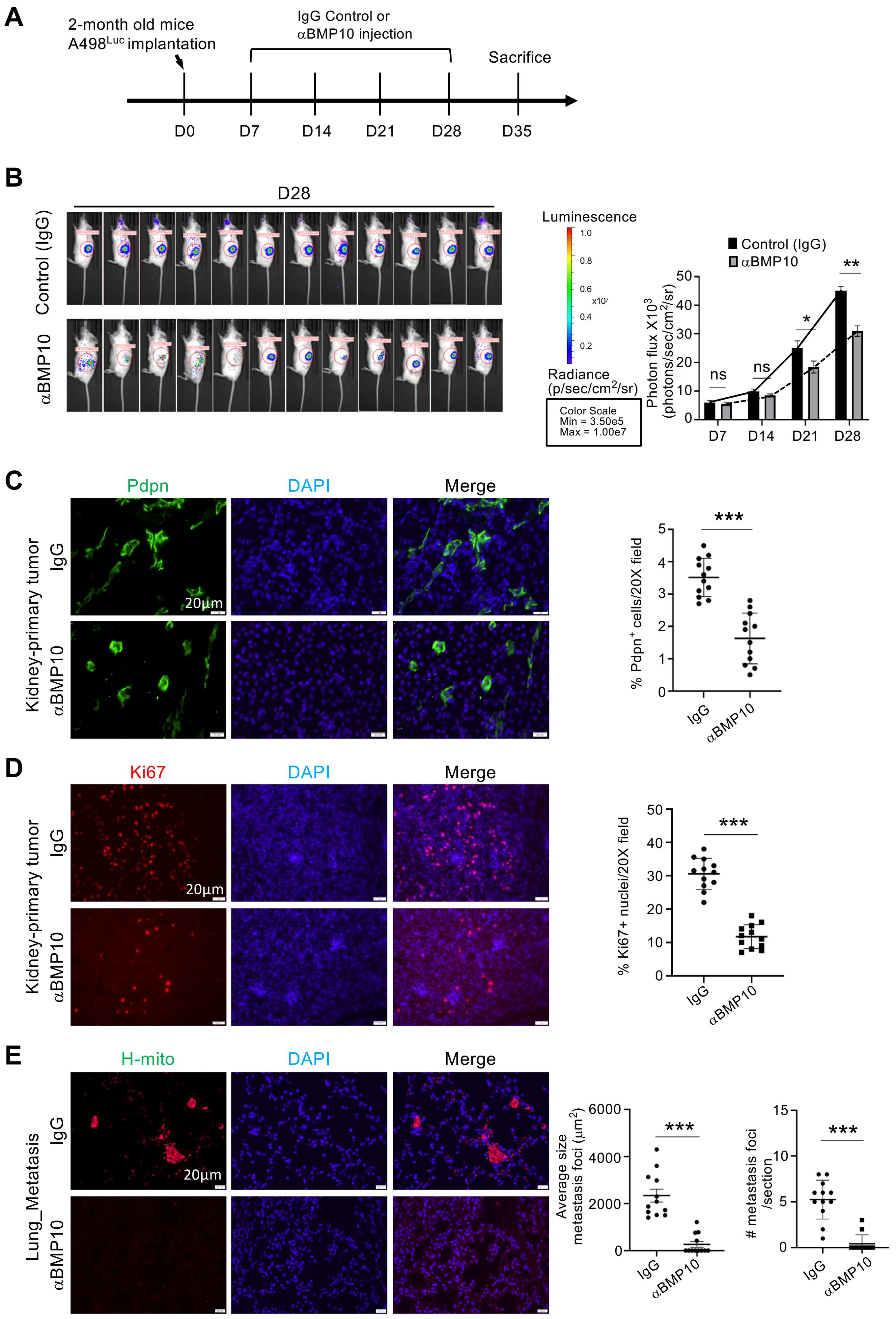
A
2-month old mice
A498Luc implantation
IgG Control or
αBMP10 injection
Sacrifice
D0
D7
D14
D21
D28
D35
B
D28
Control (IgG)
αBMP10
Luminescence
x10⁷
Radiance
(p/sec/cm²/sr)
Color Scale
Min = 3.50e5
Max = 1.00e7
Control (IgG)
αBMP10
Photon flux X10³
(photons/sec/cm²/sr)
ns
ns
*
**
D7
D14
D21
D28
C
Pdpn
DAPI
Merge
Kidney-primary tumor
IgG
αBMP10
20μm
% Pdpn+ cells/20X field
***
IgG
αBMP10
D
Ki67
DAPI
Merge
Kidney-primary tumor
IgG
αBMP10
20μm
% Ki67+ nuclei/20X field
***
IgG
αBMP10
E
H-mito
DAPI
Merge
Lung_Metatasis
IgG
αBMP10
20μm
Average size
metastasis foci (μm²)
***
metastasis foci
/section
***
IgG
αBMP10

Figure S10. BMP10 inhibition suppresses tumor progression and metastasis in vivo. **A,** Schematics of ccRCC cell implantation to the left kidney and i.v. injection of control IgG or anti-BMP10 neutralizing antibody (αBMP10). **B,** IVIS examination of xenografted mice at designated time after cancer cell implantation. The growth inhibitory effect of αBMP10 becomes obvious on D21. n=12. ns, no significance; *, *P*<0.05; **, *P*<0.01. Each data point represents one independent sample. **C,** Kidney sections from sacrificed xenografted mice on D35 were stained for Pdpn. αBMP10 treatment significantly reduces the number of $Pdpn^+$ cells of the primary tumor. Bars are 20 μm. n=12. ***, *P*<0.001. Each data point represents the average of 5 random 20x fields of views of one independent sample. **D,** Kidney sections from sacrificed xenografted mice on D35 were stained for Ki67. αBMP10 treatment significantly reduces proliferation of the primary tumor. Bars are 20 μm. n=12. ***, *P*<0.001. Each data point represents the average of 5 random 20x fields of views of one independent sample. **E,** Lung sections from sacrificed xenografted mice on D35 were stained for human mitochondria (H-mito) for visualizing metastasized ccRCC cells. αBMP10 treatment significantly reduces the size and number of metastatic foci. Bars are 20 μm. n=12. ***, *P*<0.001. Each data point represents the average of 5 random 20x fields of views of one independent sample.
