# Supplementary material for: Tumor-Induced PDPN+ Lymphatic-like Endothelial Cells Promote Clear-Cell Renal Cell Carcinoma Progression Through Reciprocal BMP10-CXCL13 Signaling
Source: Int J Mol Sci. 2026 Aug 4;27(15):6994. doi: 10.3390/ijms27156994 (PMC13467439; doi:10.3390/ijms27156994)
Supplement: Supplementary file 1 [file ijms-27-06994-s001.zip › Table S1.pdf]

Table S1. List of reagents

Reagent	Source	Catalog Number	Application/Use
Acriflavine hydrochloride	MedChemExpress	HY-B0949	HIF inhibitor
Alk1/2 inhibitor LDN-212854	Selleckchem	S7141	Alk1 inhibition in vivo
Antigen retrieval buffer - sodium citrate, pH 6	Proteintech	PR#0001	IHC, IF
Avidin/Biotin Blocking Kit	Vector	SP-2001	Block endogenous biotin
Azure Fluorescent Blot Blocking Solution	Azure	S1044	Blocking for fluorescent WB
BCA Protein Assay Kit	ThermoFisher	23225	Protein quantification
Bioanalyzer System RNA Kit	Agilent Technologies,	5067-1511	Isolated RNA quality control
Recombinant mouse BMP10 protein	R&D Systems	6038-BP	In vitro stimulation of lymphatic endothelial cells
Bovine pituitary extract (BPE)	Gibco	17005-042	Supplement for Keratinocyte SFM
CCK-8	Abcam	ab228554	Cell viability assay
Collagenase II	Sigma-Aldrich	6885	Tissue dissociation
Crystal violet	Sigma-Aldrich	C0775	Cell staining
Recombinant human CXCL13 protein	R&D Systems	801-cx-025	Cell stimulation
DAB Substrate Kit	Vector	SK-4100	IHC chromogen
Dispase	Sigma-Aldrich	D4693	Tissue dissociation
DMEM	Gibco	12100046	Cell culture
DMEM/F12	Gibco	11330032	Cell culture
DMSO (dimethyl sulfoxide)	Sigma-Aldrich	20-139	Solvent for LDN-212854
DNase I	Roche/Merck	10104159001	Tissue dissociation
CLARITY Western ECL Substrate	Bio-Rad	1705061	Western blot
EGF (for Keratinocyte SFM)	Gibco	17005-042	Supplement for Keratinocyte SFM
Endothelial cell medium	ScienCell	1001	EC growth medium
Endothelial cell growth supplement (ECGS)	Sigma-Aldrich	2759	EC growth medium supplement
Enhanced Chemiluminescence (ECL)	ThermoFisher	32106	Western blot
anti-EpCAM microbeads	Miltenyi Biotec	130-061-101	EpCAM+ cell exclusion
Evans Blue	Sigma-Aldrich	E2129	Lymphatic drainage assay
EZ-PCR Mycoplasma Detection Kit	Biological Industries	20-700-20	Mycoplasma test
FBS, heat-inactivated	Gibco	10437-028	Serum supplement
FluoroFix Buffer	BioLegend	422101	Fixation for flow cytometry
Gelatin (from bovine skin)	Sigma-Aldrich	G9391	Dish coating
Glucose	Biological Industries	41-302	Media supplement for RPMI (4.5 g/L)
GoTaq qPCR Master Mix	Promega	A6002	qPCR
Gscript First-Strand Synthesis Kit	GeneDireX	MB305-0050	cDNA synthesis
HEPES	Gibco	15630-080	Buffer supplement

High-Capacity cDNA Reverse Transcription Kit	Applied Biosystems	4368814	cDNA synthesis
Human TruStain FcX	BioLegend	422301	Fc block (human)
iTaq Universal SYBR Green Supermix	Bio-Rad	1725121	qPCR
Keratinocyte SFM	Gibco	17005-042	Cell culture (HK-2); includes EGF & BPE
lentiCRISPRv2 vector	Addgene	52961	SpCas9 expression for gene targeting
Lentivirus packaging plasmid psPAX2	Addgene	12260	Lentivirus packaging
Lentivirus packaging plasmid pMD2.G	Addgene	12259	Lentivirus packaging
L-glutamine	Gibco	25030-149	Cell culture supplement
Lipofectamine 3000 Transfection Kit	Invitrogen	L3000-015	Transfection
Low Fluorescence PVDF Membrane	Azure	S1011	WB transfer membrane
LS columns	Miltenyi Biotec	130-042-401	Magnetic beads separation
Magnetic separator	Miltenyi Biotec	130-092-168	Magnetic beads separation
Matrigel Basement Membrane Matrix (Growth factor reduced)	Corning	354230 / 356230	Invasion assay, cancer cell implantation
MEM	Gibco	41500-034	Cell culture
Methanol	Genestar	AM0440-4000	Fixation
Micromount Mounting Medium	Leica	3801731	Mounting medium
Mouse TruStain FcX PLUS	BioLegend	156603	Fc block (mouse)
Neutral Buffered Formalin	Leica	3800600	Fixative
Normal Goat Serum	Jackson ImmunoResearch	AB2336990	Blocking serum
PEG300	Sigma-Aldrich; 807484	807484	Solvent for LDN-212854
Penicillin–Streptomycin	Gibco	10378016	Antibiotics
Permeabilization Buffer	BioLegend	421002	Permeabilization for IHC staining
Phosphatase Inhibitor Cocktail 3	Sigma-Aldrich	P0044-1ML	Phosphatase inhibition
Pierce BCA Protein Assay Kit	ThermoFisher	23225	Protein quantification
ProLong Gold Antifade	ThermoFisher	P36935	Mounting antifade reagent
Puromycin	InvivoGen	ant-pr-1	Selection antibiotic
RIPA Lysis Buffer	ThermoFisher	89901	Protein extraction
RNA Isolation Kit	GeneDireX	NA017-0100	RNA extraction
RPMI 1640	Gibco	31800-022	Cell culture (786-O, HK-2)
Sodium pyruvate	Gibco	11360-070	Cell culture supplement
SYBR Green Master Mix	Bio-Rad	4913914001	PCR
TRIzol reagent	Invitrogen	15596026	RNA isolation
TrueVIEW Autofluorescence Quenching Kit	Vector	SP-8400	Reduce autofluorescence
Trypan blue	ThermoFisher	15250061	Cell viability test

TrypLE Express Enzyme (1X), phenol red	Gibco	12605010	Cell dissociation (animal-origin free)
Trypsin–EDTA (0.25%)	Gibco	15400-054	Cell dissociation
Tween-80	Sigma-Aldrich	822187	Solvent for LDN-212854
Ultra-LEAF Purified anti-human BMP-10 (clone A17182F)	BioLegend	618104	Neutralization of human BMP-10 in vitro (validated in ATDC5 cells)
Vectastain Elite ABC Kit	Vector	PK-6100	Signal amplification
