# Supplementary material for: Tumor-Induced PDPN+ Lymphatic-like Endothelial Cells Promote Clear-Cell Renal Cell Carcinoma Progression Through Reciprocal BMP10-CXCL13 Signaling
Source: Int J Mol Sci. 2026 Aug 4;27(15):6994. doi: 10.3390/ijms27156994 (PMC13467439; doi:10.3390/ijms27156994)
Supplement: Supplementary file 1 [file ijms-27-06994-s001.zip › Table S2.pdf]

Table S2. List of antibodies

Antibody	Source	Reactivity	Catalog #	Application	Dilution
Rabbit monoclonal anti-β-Actin	Cell Signaling; RRID:AB_2223172	Most species	4970	WB	1:1000
Rat monoclonal anti-β-Actin	Biolegend; RRID:AB_2721349	Most species	664802	WB	1:1000
Rabbit monoclonal anti-Akt	GeneTex	Human, mouse, rat	HL2915	IHC, WB	1:1000
Rabbit monoclonal anti-AKT (phospho Ser473)	GeneTex	Human, mouse	HL2971	WB	1:1000
Polyclonal goat anti-human ALK-1 antibody	R&D Systems	Human, mouse	AF370	IHC	1:100
Mouse monoclonal anti-BMP10 (clone 462732)	R&D Systems	Human, mouse	MAB2926	Neutralizing	5mg/kg
Rat monoclonal anti-Bmp10	R&D Systems	Mouse	MAB6038	IHC	1:100
Ultra-LEAF Purified anti-human BMP10 (clone A17182F)	BioLegend	Human	618104	Neutralizing antibody in xenograft	5 mg/kg
Mouse monoclonal anti-CA9/CAIX	Santa Cruz	Human	sc-365900	IHC	1:100
Alexa Fluor 488 mouse monoclonal anti-CA9 (CAIX)	Santa Cruz	Human	sc-365900	IHC	1:100
Rat monoclonal anti- Cd45	Biolegend; RRID:AB_2563541	Mouse	103129	FC	1:50
Rat monoclonal anti-Cd45-PerCP	Biolegend	Mouse	103132	FC	1:50
Goat polyclonal anti- Cxcl13	R&D Systems	Mouse	AF470	WB, IHC	1:1000
Rabbit polyclonal anti-CXCL13	Invitrogen	Human, mouse	PA5-79106	WB, IHC	1:1000
Goat polyclonal anti- Cxcl13	Invitrogen	Mouse	PA5-47018	WB, IF	1:1000
Rabbit monoclonal anti-CXCR5	Abcam	Human, mouse, rat	ab254415	IHC, IF, WB, FC	1:1000
Rat monoclonal anti- Cxcr5-FITC	Biolegend	Mouse	145519	FC	1:50
Mouse monoclonal anti-CXCR5	R&D Systems	Human	MAB190	IHC	1:100
Rat monoclonal anti- Cxcr5	R&D Systems	Mouse	MAB6198	IHC	1:100
Rabbit polyclonal anti-CXCR5	GeneTex	Human	GTX100351	IHC, IF, WB	1:1000
Goat polyclonal anti- GFP	GeneTex	Universal	TX26673	IHC, IF	1:100
Rabbit monoclonal anti-GFP	Cell Signaling; RRID:AB_1196615	Universal	2956	IHC, IF	1:200
Goat polyclonal anti-GFP	GeneTex	Universal	GTX26673	IHC	1:1000
Rabbit monoclonal anti-Ki67	Cell Signaling	Mouse	12202	IHC, IF	1:100
Rabbit polyclonal anti-Ki67	Abcam; RRID:AB_443209	Human, mouse	ab15580	IHC, IF	1:100
Rat monoclonal anti-Lyve1-Alexa Fluor 488	ThermoFisher	Mouse	53-0443-82	IHC, FC	1:50
Goat polyclonal anti- Lyve1	R&D Systems	Human	AF2089	IHC	1:100
Mouse monoclonal anti-mitochondria	Millipore; RRID:AB_94052	Human	MAB1273	IHC	1:100
Rabbit polyclonal anti-Nephrin-FITC	Biorbyt	Human, mouse	orb189951	FC	1:50

Rabbit polyclonal anti-PDPN	Proteintech	Human	11629-1-AP	IHC	1:100
Rat monoclonal anti-Pdpn	Abcam	Mouse	ab256559	IHC, WB	1:1000
Rat monoclonal anti-mouse Pdpn-Alexa Fluor 488	Biolegend	Mouse	156207	IHC, IF (3D culture)	1:100
Rabbit polyclonal anti-PDPN	Abcam	Human, mouse	ab109059	WB, IF, IHC	1:1000
Sheep polyclonal anti-PDPN	R&D Systems	Human	AF3670	IHC	1:100
Syrian hamster monoclonal anti-Pdpn-APC	Biolegend	Mouse	127410	FC	1:50
Rabbit polyclonal anti-PROX1	Biolegend	Human, mouse, rat	925202	IHC, IF, WB	1:1000
Rabbit monoclonal anti-PROX1	Abcam	Human, mouse, rat	ab199359	IHC	1:1000
Goat polyclonal anti-VE-Cadherin	R&D Systems	Mouse	AF1002	IHC, IF	15 □g/ml
Rabbit polyclonal anti-VEGFR3	Abcam	Human, mouse, rat	ab27278	IHC, WB	1:1000
Goat polyclonal anti-Vegfr3	R&D Systems	Mouse	AF743	IHC	1:100
Rat monoclonal anti-Vegfr3	Novusbio	Mouse	RM0003-5F63	WB, IF, IHC	1:1000
Rabbit polyclonal anti-VEGFR3	Arigo	Human, mouse, rat	ARG58698	FC, IF, IHC, WB	1:1000
Rabbit polyclonal anti-VHL	Cell Signaling	Human	68547S	WB	1:1000
Goat anti-mouse IgG-Alexa Fluor 568	ThermoFisher	-	A11004	Secondary	1:200
Goat anti-mouse IgG-Alexa Fluor 647	ThermoFisher	-	A21235	Secondary	1:200
Goat anti-rabbit IgG-Alexa Fluor 488	ThermoFisher	-	A32731	Secondary	1:200
Goat anti-rat IgG-Alexa Fluor 647	ThermoFisher	-	A21247	Secondary	1:200
Donkey anti-goat IgG-Alexa Fluor 488	Abcam	-	ab150129	Secondary	1:200
Goat polyclonal anti-mouse IgG-HRP	Jackson Immunoresearch; RRID:AB_2338504	-	115-035-062	Secondary	1:200
Goat polyclonal anti-rabbit IgG-HRP	Jackson Immunoresearch; RRID:AB_2313567	-	111-035-003	Secondary	1:200
Azure Spectra 700 goat anti-mouse	Azure; RRID:AB_3331665	-	S1015	Secondary	1:3000
Azure Spectra 800 goat anti-rabbit	Azure; RRID:AB_3331663	-	S1021	Secondary	1:3000
Donkey anti-goat IgG-Alexa Fluor 488	Invitrogen	-	A11055	Secondary	1:100
Donkey anti-mouse IgG-Alexa Fluor 488	Invitrogen	-	A21202	Secondary	1:100
Donkey anti-mouse IgG-Alexa Fluor 647	Invitrogen	-	A31571	Secondary	1:50
Donkey anti-mouse IgG-Alexa Fluor 568	Invitrogen	-	A10042	Secondary	1:200
Donkey anti-goat Goat IgG-Alexa Fluor 488	ThermoFisher	-	A32814	Secondary	1:200
Donkey anti-goat IgG-Alexa Fluor 488	ThermoFisher	-	A11057	Secondary	1:200
Donkey anti-goat IgG-Alexa Fluor 647	ThermoFisher	-	A32849	Secondary	1:200

Mouse IgG1 κ isotype control-Alexa Fluor 700	Biolegend	-	400143	FC	1:50
Rat IgG2b κ isotype control-FITC	Biolegend	-	400633	FC	1:50
Mouse IgG2a κ isotype control-PerCP	Biolegend	-	400529	FC	1:50
Mouse IgG1 κ isotype control-PE	Biolegend	-	400111	FC	1:50
Rat IgG2b κ isotype control-PE	Biolegend	-	400607	FC	1:50
Rat IgG2a κ isotype control-APC	Biolegend	-	400511	FC	1:50
Rat IgG2b κ isotype control-PE	Biolegend	-	103105	FC	1:50
Syrian hamster IgG control-APC	Biolegend	-	402012	FC	1:50

FC, Flow cytometry; IF, Immunofluorescence; IHC, Immunohistochemistry; WB, Western blotting
