# Supplementary material for: Tumor-Induced PDPN+ Lymphatic-like Endothelial Cells Promote Clear-Cell Renal Cell Carcinoma Progression Through Reciprocal BMP10-CXCL13 Signaling
Source: Int J Mol Sci. 2026 Aug 4;27(15):6994. doi: 10.3390/ijms27156994 (PMC13467439; doi:10.3390/ijms27156994)
Supplement: Supplementary file 1 [file ijms-27-06994-s001.zip › Table S3.pdf]

Table S3. qRT-PC R primers

Gene	Forward primer	Reverse primer
Acta2 (αSMA)	TGCTGACAGAGGCACCACTGAA	CAGTTGTACGTCCAGAGGCATAG
Col1a1	CCTCAGGGTATTGCTGGACAAC	CAGAAGGACCTTGTTTGCCAGG
Pdgfrb	GTGGTCCTTACCGTCATCTCTC	GTGGAGTCGTAAGGCAACTGCA
Pdpn	ACAACCACAGGTGCTACTGGAG	GTTGCTGAGGTGGACAGTTCCT
Lyve1	ACCAGGTAGAGTCAGCGCAGAA	CAGGACACCTTTGCCATTCTTCC
Flt4 (Vegfr3)	AGACTGGAAGGAGGTGACCACT	CTGACACATTGGCATCCTGGATC
Acvrl1 (Alk1)	CTTCTTGGTGCAGAGGACGGTA	CGTGAGGAGAAAATCTTGACCGC
Cxcl13	CATAGATCGGATTCAAGTTACGCC	GTAACCATTTGGCACGAGGATTC
Actb (β-actin)	CATTGCTGACAGGATGCAGAAGG	TGCTGGAAGGTGGACAGTGAGG
